# Current Insight of Peptide-Based Hydrogels for Chronic Wound Healing Applications: A Concise Review

**DOI:** 10.3390/ph18010058

**Published:** 2025-01-07

**Authors:** Aifa Asyhira Khairul Nizam, Syafira Masri, Nur Izzah Md Fadilah, Manira Maarof, Mh Busra Fauzi

**Affiliations:** 1 Department of Tissue Engineering and Regenerative Medicine, Faculty of Medicine, Universiti Kebangsaan Malaysia, Cheras, Kuala Lumpur 56000, Malaysia; p138495@siswa.ukm.edu.my (A.A.K.N.); syafiramasri@ukm.edu.my (S.M.); izzahfadilah@ukm.edu.my (N.I.M.F.); manira@ukm.edu.my (M.M.); 2 Advance Bioactive Materials-Cells UKM Research Group, Universiti Kebangsaan Malaysia, Bangi 43600, Selangor, Malaysia; 3 Ageing and Degenerative Disease UKM Research Group, Universiti Kebangsaan Malaysia, Bangi 43600, Selangor, Malaysia

**Keywords:** peptide-based hydrogel, chronic wound, biodegradation, biocompatibility

## Abstract

Chronic wounds present a substantial healthcare obstacle, marked by an extended healing period that can persist for weeks, months, or even years. Typically, they do not progress through the usual phases of healing, which include hemostasis, inflammation, proliferation, and remodeling, within the expected timeframe. Therefore, to address the socioeconomic burden in taking care of chronic wounds, hydrogel-based therapeutic materials have been proposed. Hydrogels are hydrophilic polymer networks with a 3D structure which allows them to become skin substitutes for chronic wounds. Knowing that peptides are abundant in the human body and possess distinct biological functionality, activity, and selectivity, their adaptability as peptide-based hydrogels to individual therapeutic requirements has made them a significant potential biomaterial for the treatment of chronic wounds. Peptide-based hydrogels possess excellent physicochemical and mechanical characteristics such as biodegradability and swelling, and suitable rheological properties as well great biocompatibility. Moreover, they interact with cells, promoting adhesion, migration, and proliferation. These characteristics and cellular interactions have driven peptide-based hydrogels to be applied in chronic wound healing.

## 1. Introduction

A wound is a breakage or defect in the skin caused by physical or thermal damage, as well as underlying medical or physiological disorders that can disrupt normal body processes. Healthy skin primarily consists of three layers: the epidermis, dermis, and subcutaneous layer [1]. The epidermis serves as the outermost protective barrier. Due to its great impermeability, the epidermis is able to efficiently regulate water loss and serve as a protective barrier against external irritants. The dermis is a layer of connective tissue that provides support to the epidermis. It is composed of extracellular matrix proteins such as collagens, elastin, proteoglycans, and glycosaminoglycans, which are produced by fibroblasts [2]. The subdermal layer contains a significant amount of adipose tissue, which is well supplied with blood vessels. This allows for effective regulation of skin temperature and contributes to the mechanical qualities of the skin. Understanding the composition of normal skin is beneficial for comprehending the process of wound healing [3].

**Figure 1:**
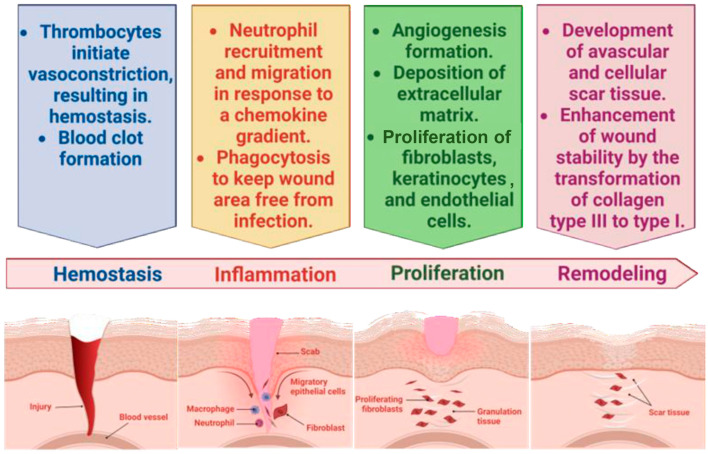
Wound healing pathophysiology presents a comprehensive outline of the consecutive or concurrent phases implicated in the process of human skin wound healing, commencing with the original trauma, concluding with the tissue remodeling, and the processes involved in each phase of wound healing: hemostasis, inflammation, proliferation, and remodeling. Image created by using Biorender.com.

Briefly, acute and chronic skin wounds are distinguished by their pathophysiology and consequences. Acute wounds experience a sequence of molecular processes that ultimately lead to the restoration of their structural integrity. In contrast, chronic wounds do not heal and are characterized by pathological processes, such as ongoing inflammation, persistent infections, and tissue death [4]. The process of acute wound healing can be divided into four distinct phases: hemostasis, inflammation, proliferation, and remodeling. Hemostasis is the initial response to an acute wound, as it stops bleeding and prevents blood loss. During the inflammatory phase, the skin’s injury triggers a complex immune response that eliminates the pathogens that penetrate the wound and prepares the tissue for the restoration of anatomical integrity. However, in chronic wounds, there is a prolonged and ongoing inflammatory response that results in a significant buildup of reactive oxygen species (ROS) [5]. This buildup exceeds the cells’ ability to counteract it with antioxidants, which in turn inhibits the transition of the wound from the inflammatory phase to the proliferative phase [6]. Figure 1 shows a comprehensive outline and illustration of the wound healing process.

The hemostasis phase of wound healing starts with the activation of platelets, which produce the substances accountable for the production of fibrin clots. This process restores the local cessation of bleeding and serves as a temporary extracellular matrix for the movement of blood cells. The exudate containing coagulation factors will undergo coagulation at the location of the lesion, therefore offering mechanical support to the damaged tissue [3]. During the inflammatory stage, cellular components such as platelets, lymphocytes, monocytes, neutrophils, and macrophages are responsible for clearing away debris and preparing the wound site for the development of granulation tissue [3]. Platelets and injured cells release cytokines and growth factors, including IL-1β (interleukin-1β), TNF-α (tumor necrosis factor-α), FGF (fibroblast growth factor), and PDGF (platelet-derived growth factor), which attract leukocytes to the wound site. Platelets are the primary source of PDGF; nevertheless, macrophages, fibroblasts, and endothelial cells can also release PDGF, especially during wound healing and inflammation.

During the proliferative stage, injured and destroyed tissues are regenerated by the growth of epithelial cells and fibroblasts. As a result, the open wound eventually gets filled with granulation cells. The granulation tissue is synthesized, and cells undergo strong migration and proliferation. This tissue is composed of macrophages, endothelial cells, fibroblasts, and provisional extracellular matrix. Although injured cells may release early signaling molecules that begin the wound healing process, macrophages and fibroblasts serve as the main sources of essential growth factors including VEGF, FGF, and TGF-β1, which facilitate angiogenesis, fibroblast proliferation, and tissue repair. Fibroblasts produce several components of the provisional extracellular matrix, including type III collagen, proteoglycans, and fibronectin, to facilitate the movement of cells into the region [3,7,8]. The final stage, maturity or remodeling, is characterized by the formation of connective tissue and the enhancement of new epithelium. It is important to acknowledge that these periods are not rigorously and explicitly differentiated by time period, but rather are overlapping [3]. The wound healing process concludes with the period of tissue maturation and remodeling, which is facilitated by the degradation of type III collagen and its replacement by type I collagen. This alteration to collagen type I improves the ability of the tissue to resist tension, hence increasing its tensile strength [9].

Previously, chronic wounds have been treated using autografts, allografts (generally acquired from cadavers), or xenografts (often extracted from pig skin). Autografts are tissue grafts harvested from the same individual [10] to restore damaged or missing tissue, providing the benefits of biocompatibility and a reduced chance of rejection. They are commonly utilized for skin regeneration due to their low risk of immunological rejection. Allografts are tissue transplants obtained from a genetically different donor of the same species [10]. Allografts have historically served as conventional skin substitutes for patients with significant burns, providing a temporary skin covering [11]. The adaptability and availability of allografts facilitate the prompt and efficient excision of extensive burns. However, allografts may experience immunological rejection, necessitating immunosuppressive medication to avert this outcome. Xenografts refer to tissue grafts obtained from a different species [10], such as swine or bovine tissues. Xenografts can effectively repair acute severe wounds, such as diabetic foot ulcers, because to their preservation of the collagenous structure after decellularization and their derivation from diverse sources, including porcine, bovine, and fish origins [12]. Autologous skin grafts can be classed based on their anatomical structure as epidermal skin grafts (ESGs), split-thickness skin grafts (STSGs), and full-thickness skin grafts (FTSGs) [13]. Skin grafts are frequently used to treat chronic wounds, especially when the wound is extensive, profound, or does not respond to traditional treatments.

Although skin grafts can be efficacious, they also have many drawbacks that might influence the overall efficacy of the treatment and patient outcomes. Although there have been many advancements in wound therapies, a significant percentage of leg ulcers (25% to 50%) and foot ulcers (>30%) remain unhealed even after 6 months of treatment [14]. These injuries may be appropriate for undergoing skin transplantation. Nevertheless, the occurrence of a new wound at the location where tissue is taken carries other accompanying medical conditions, such as discomfort, formation of scars, and susceptibility to infection, which provide notable disadvantages to this particular method [13]. Despite extensive research, autologous skin grafting, specifically split-thickness skin grafting, remains the main approach used by reconstructive surgeons to treat chronic skin injuries. However, there are a few restrictions and adverse health consequences associated with the area from which the skin is taken, resulting in complications that may pose significant obstacles and a decrease in aesthetic appearance, particularly in individuals with extensive skin damage [15]. Achieving permanent wound closure with a satisfactory aesthetic outcome is also difficult when treating large skin injuries. To minimize the socioeconomic burden of taking care of chronic wounds and taking into consideration the anticipated rise in expenditures resulting from demographic changes, it is crucial to prioritize achieving full wound closure immediately [16].

Moreover, it is commonly understood that in many skin conditions, such as severe burns or chronic wounds, large amounts of medications supplied throughout the body, such as antibiotics, are necessary to accomplish desired healing effects in the affected area. Systemic antibiotics have been used to enhance symptom control and mitigate the progression of deterioration and infections. Nevertheless, the efficacy of systemic antibiotics in penetrating wound biofilms is limited [17]. Therefore, employing an antisepsis strategy is a more effective approach for the treatment and prevention of bacterial infections in wounds. Conventional wound dressings are considered passive and are typically used on wounds that are dry and thoroughly cleansed [18]. These dressings include gauzes and bandages. Gauze dressings are composed of cotton fibers, viscose, and polyesters, which can be either woven or non-woven. The primary purpose of these dressings is to absorb exudate and fluid from an open wound. An instance of this is Xeroform™, a non-occlusive dressing consisting of petrolatum gauze infused with 3% bismuth tribromo phenate [19]. It is employed to provide coverage for moderate wounds that are either dry or exudative. However, standard wound dressings may not be suited for chronic wounds due to their limited drainage capabilities and tendency to stick to the wound surface, making it challenging to replace the dressing [20].

**Figure 2:**
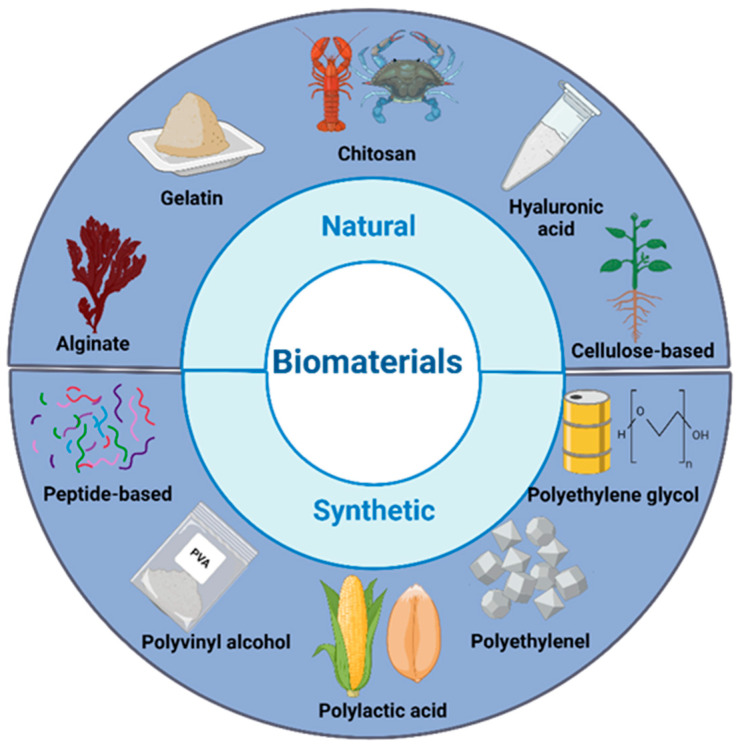
Classification of biomaterials for wound healing. This figure illustrates the diverse array of biomaterials employed in wound healing applications, encompassing both natural sources derived from living organisms and synthetic materials engineered through chemical processes. Image created by using Biorender.com.

Hydrogels are polymer networks that are hydrophilic and 3D, allowing them to hold significant quantities of water. This property makes them very appropriate for use in wound healing. Hydrogels are 3D structures made up of hydrophilic polymers that are connected by either physical or chemical crosslinked connections. The hydrophilic structures that cannot be dissolved display a remarkable ability to collect fluids released from wounds and facilitate the absorption of oxygen, therefore accelerating the healing process [21]. As previously mentioned, hydrogels are characterized by a 3D polymeric network that is highly hydrated. They have the ability to bind a significantly larger amount of water relative to their dry weight [22]. This property allows them to keep the wound bed consistently wet. Because of their distinctive physical qualities, hydrogel networks may be molded into different sizes and forms [23]. In addition, hydrogels also provide a platform for incorporating cells, antibacterial agents, growth hormones, and multiple additional biomolecules. As a result, hydrogel-based materials are the most appropriate skin substitutes for treating chronic wounds [24]. Figure 2 shows the types of biomaterials, natural and synthetic, which are employed in wound healing applications.

Considering extracellular matrix (ECM) similarities, hydrogels utilized for wound healing purposes must offer a cell-compatible 3D environment that facilitates tissue regeneration. It is crucial for hydrogels to fulfil the fundamental criteria of being biocompatible for therapeutic usage and to also have certain physical and mechanical characteristics that are suitable for treating skin wounds. Furthermore, it is necessary for them to provide a suitable environment for the formation of blood vessels and the proliferation of cells. Moreover, biodegradability is the capacity of a material to decompose in response to its interaction with a biological environment. In the context of wound recovery, the rate of hydrogel biodegradation is crucial for the repair and regeneration of chronic lesions, particularly those that are deeper [25]. Hence, natural polymers are commonly used for biomedical applications due to their key attributes such as excellent biocompatibility and great biodegradability.

Protein-based polymers derived from the extracellular matrix (ECM), including gelatin and collagen, are frequently employed to produce biodegradable hydrogels. Gelatin is a hydrophilic polymer that is derived from collagen through a process of hydrolysis and denaturation at high temperatures. Various abundant sources may be utilized for the extraction of collagen and gelatin [26]. Gelatin possesses several benefits as a wound treatment, such as its great solubility and ease of extraction and synthesis [27]. These biomaterials are beneficial because their inherent cell recognition molecules promote cell attachment and regulated enzymatic biodegradation when cells multiply and tissues undergo remodeling [28]. Collagen and gelatin have the ability to form hydrogels by physical crosslinking when there is a change in temperature. Gelatin is a well-established biopolymer that is used in a variety of biological applications due to its relatively low antigenicity. However, gelatin is unable to maintain its whole hydrogel structure at temperatures that are typical of the human body. Nevertheless, gelatin is a hydrophilic protein, and crosslinking is typically necessary to improve its mechanical performance and stability. Consequently, gelatin materials are insoluble in biological environments [29]. Chemical crosslinking methods that are compatible with cells are frequently employed to enhance the stability of hydrogels made from gelatin [28]. The rapid degradation of gelatin-based hydrogels can be a disadvantage; however, the degradation behavior during the wound healing process can be customized by increasing the concentration of gelatin and the degree of crosslinking between polymer chains [30].

Moreover, there are a variety of gelatin crosslinking procedures available, including enzymatic processes using transglutaminase and chemical processes using fructose, diepoxy, genipin, dextran dialdehyde, formaldehyde, diisocyanates, glutaraldehyde, or carbodiimide, respectively [31]. Furthermore, gelatin-based hybrid hydrogels have mechanical characteristics that closely resemble those of human skin, indicating their potential compatibility with the skin [32]. Hybrid hydrogels are 3D polymeric networks that integrate two or more distinct materials to achieve outstanding characteristics. These hydrogels often combine natural and synthetic polymers, enabling customized mechanical, chemical, and biological properties appropriate for many biomedical applications, including tissue engineering and drug delivery. The combination of materials may increase bioactivity, promote stability, and regulate the release kinetics of therapeutic drugs. Hybrid hydrogels have been created to mimic the molecular structures, dynamic responsiveness, and specific biological activities of natural proteins, while including the tunability and processability afforded by synthetic polymer components [33]. Researchers have documented fascinating characteristics of gelatin-based hybrid hydrogels through a variety of in vitro and in vivo tests. Due to their intriguing characteristics, they have great potential as scaffolds for wound dressings. The incorporation of natural polymers with other polymers (particularly synthetic polymers) in the production of hydrogels has led to a superior mechanical performance [34,35]. This is advantageous for the convenient manipulation and use of hydrogels in wound care.

As an example, chitosan possesses structural properties that closely resemble those of the extracellular matrix. This similarity promotes the growth, organization, and migration of cells during tissue remodeling [36]. Furthermore, chitosan possesses several active functional groups that are capable of binding to proteins. Additionally, its positive charge may efficiently stimulate cell interaction and differentiation. In addition, cellulose derivatives have the capacity and optimal application to create innovative wound dressings that can improve wound healing, acquired by replacing the hydroxyl groups in the cellulose molecule with various alkyl groups [37]. They can maintain optimal local moisture levels and are highly effective at absorbing and holding onto a significant amount of wound exudate in the interstitial regions of their networks. Additionally, cellulose’s wound healing efficacy is derived from its capacity to expedite the wound healing process by incorporating several growth factors at the site of damage, including basic fibroblast growth factor, phosphodiesterase growth factor, and epidermal growth factor. These growth factors are not naturally present in cellulose but can be added separately, and may able to stimulate the migration and proliferation of skin cells called dermal fibroblasts, while also accelerating the wound healing process. Cellulose-based materials have demonstrated the capacity to improve wound healing by incorporating bioactive agents [38], providing a favorable environment for biological functions, including the proliferation, migration, and differentiation of fibroblasts and keratinocytes.

Alginate is a type of biopolymer made up of polysaccharides. It is well recognized as a naturally occurring biopolymer used in wound treatment due to its biocompatibility, ability to form gels, and tendency to swell [39,40]. These properties provide a moist microenvironment at the wound site, which promotes effective healing and reduces the time required for healing. Calcium alginate, among the several salt forms of alginate, has the natural capacity to enhance blood clotting by activating platelets by the release of calcium ions at the site of the injury [41]. Calcium alginate dressings establish a protective barrier that facilitates exudate absorption and reduces moisture around the injury area. This barrier function reduces the likelihood of infection by preventing bacterial invasion. Upon the application of calcium alginate-based wound dressings to the wound site, these dressings maintain a moist environment, minimize bacterial infection, and expedite the wound healing process. Nowadays, alginate is a desirable material for wound healing treatment due to its hydrophilicity and substantial liquid-absorbing capacity. Alginate hydrogels are superior to typical medical dressings due to their non-toxic nature and high water absorption capacity, and they have the ability to create a hydrogel network on the wound surface, which helps maintain a moist environment [42]. Hyaluronic acid (HA), a key constituent of the extracellular matrix, promotes wound vascularization, epithelialization, and collagen deposition. During the proliferation phase, hyaluronic acid (HA) specifically promotes the migration and proliferation of fibroblasts and keratinocytes [43]. Furthermore, the tendency of HA to absorb water ensures that wound hydration is preserved, and it is non-antigenic. These biological functions, which produce unique characteristics in comparison to other materials, lead to an advantageous acceleration of wound recovery [44]. Nevertheless, HA requires chemical functionalization to introduce crosslinking sites, as it does not form hydrogels on its own due to its weak mechanical strength and stability. Chemical modifications (such as methacrylate, tyramine, and more) target the active hydroxyl and carboxyl groups that are present in the HA chain [45]. This modification is often useful prior to crosslinking formation as it enhances HA’s stability in aqueous solutions [46].

The functioning and efficacy of wound dressings are greatly influenced by the properties of the polymers, especially their rheological and swelling characteristics, which are crucial for wound care. Viscosity and elasticity are examples of rheological characteristics that influence how a polymer-based dressing can be applied to a wound and how efficiently it fits to irregular wound surfaces, providing better coverage and stability. Swelling properties represent the ability of the scaffolds to absorb water, which is essential for absorbing excessive exudate from the wound site [47]. For instance, polymers with good swelling properties such collagen have the ability to absorb water and promote cellular infiltration and the creation of granulation tissue. Therefore, a drastic decrease in exudation and swelling has been observed, which collagen can use to help restore equilibrium in chronic wounds, as well as sped up the healing process [48]. In similar, gelatin can absorb significant amounts of water (in the range of 89–93%), which helps maintain a moist wound environment to accelerate the wound healing process [32] which is essential for optimal healing.

Furthermore, composites are typically composed of two or more materials with two or more phases with heterogenous characters. Nanocomposites are a class of materials consisting of one or more phases with nanoscale dimensions that are embedded in a matrix made of polymer, metal, or ceramic [49]. The use of composites has demonstrated significant promise in increasing the outcome of wound healing. Comparing composite polymers to single-component polymers, composite polymers offer enhanced mechanical strength, bioactivity, and controlled degradation [50,51]. This can be seen in hydrogels typically made from a single polymer network, which frequently lack the mechanical and biological characteristics needed for tissue engineering applications [51]. For example, hydrogels that are reinforced with bioactive glass or ceramic materials can provide structural support while stimulating the cellular activity that is required for tissue regeneration; this is due to their overall chemical structure, which affects their interaction with body systems, as well as the interaction of the glass surface with live tissue [52,53]. As such, they provide a viable option for advanced wound care treatments. 

This review explores the emerging role of peptide-based hydrogels in chronic wound healing, focusing on their biocompatibility, biodegradability, physicochemical and mechanical properties, as well as their therapeutic potential. The novelty of this review lies in synthesizing recent findings to provide a comprehensive understanding of the preclinical efficacy of peptide-based hydrogels. Also, the rationale for this review is to address the existing gap in the literature by highlighting the unique advantages of peptide-based hydrogels over traditional treatments and exploring their potential to improve chronic wound healing outcomes.

## 2. Peptide-Based Hydrogels for Chronic Wound Healing

The adaptability of peptide-based hydrogels to individual therapeutic requirements has made them a significant potential biomaterial for the treatment of chronic wounds. Nowadays, peptide-based wound dressings have attracted significant interest. Peptides are abundant in the human body and possess a distinct biological functionality, activity, and selectivity. As a result, both naturally occurring and chemically synthesized peptide molecules display a diverse array of biological effects [54]. Peptide-based hydrogels are becoming more attractive as biomaterials for the treatment of chronic wounds because of their distinctive characteristics that may be customized to meet specific therapeutic requirements. For instance, some peptides (such as OA-GL12, OA-GL21, RL-QN15, and Ot-WHP) obtained from the skin secretions of amphibians expedite the process of wound healing, including both acute wounds and chronic wounds, in individuals with diabetes [55].

Currently, peptide-based hydrogels have the benefits of being cost-effective, easily prepared, and having a wide range of structural variations, making them suitable for use in biomedical applications. A study on the safety assessment of peptide-based hydrogels conducted via an in vivo study showed an apparent toxicity and high biocompatibility once biodegraded [56]. The breakdown rate of peptide-based hydrogels may be precisely controlled by adjusting the monomer design and the quantities of enzymes, such as trypsin and α-chymotrypsin, which catalyze the hydrolysis of amide bonds [57]. By taking these effects into consideration, it is possible to develop biodegradable peptide-based hydrogels that possess pH-responsive properties for the purpose of drug release and wound dressing.

### 2.1. Peptide Selection: Criteria for Selecting Peptides, Including Sequence Specificity, Functional Motifs, and Bioactivity

Two decades ago, the self-assembly of small molecules to form hydrogels was a result of serendipitous observations. Nevertheless, there has been a substantial improvement in the fundamental comprehension of the self-assembly processes over the years. By taking these effects into consideration, it is possible to develop biodegradable peptide-based hydrogels that possess pH-responsive properties for the purpose of drug release and wound dressing. The hierarchical self-assembly model, understanding non-covalent interactions, and hydrophilic–lipophilic balance (HLB) offer a platform for effectively designing and preparing hydrogels [58]. Proteins and peptides may adopt numerous secondary structures, such as β-pleated sheets, α-helices, coiled–coils, and β-turns, by utilizing distinct supramolecular forces [59]. Small peptides can also exhibit secondary structures, such as organized groupings. These structures then combine in a hierarchical manner to create more complex aggregates, such as hydrogels. 

In order to construct a peptide-based hydrogel, there is a need to identify the primary sequence that will ultimately result in a secondary structure that is capable of initiating hydrogelation. This is due to certain conditions in which the probable secondary structure of a peptide is determined by its primary sequence. β-pleated sheets are the most prevalent option in this regard. A multitude of peptide hydrogelators that form β-sheets have been documented in the literature. A previous study proposed a design principle for β-sheet-type aggregates in solution that includes the following: (1) cross-strand attractive forces between side chain functionalities, (2) the adjacent β-strands must recognize laterally for one-dimensional self-assembly that evades heterogeneously aggregated β-sheet structures, and (3) the strong adhesion of water to the surface of the sheets [60]. Nevertheless, the secondary structure is not always predictable from the primary structure alone and instead depends on a variety of additional factors, including environmental factors like pH and temperature as well as solvent, ion, and cofactor interactions and how molecules interact with one another.

#### Peptide Sequence Specificity

The complex and frequently impaired healing environment renders the criteria of sequence specificity, functional motifs, and bioactivity even more critical when selecting peptides for chronic wound healing. Peptide sequences can self-assemble into supramolecular structures with weak intramolecular and intermolecular interactions. Well-designed self-assembling peptides offer a wide range of roles, such as imitating the extracellular matrix (ECM), stimulating both humoral and cellular immunity, and aiding in drug administration and targeting. In addition, hydrogelators based on low molecular weight peptides have superior biocompatibility and biodegradability compared to traditional polymer gels [61]. These hydrogelators are rarely associated with severe adverse effects and fulfil the criteria for the majority of tissue engineering applications. Consequently, self-assembling peptide-based hydrogels have a wide range of applications in nanotechnology and biomedicine, such as topical drug delivery, tumor treatment, immune adjuvants, 3D tissue cell culture, tissue repair, and tissue regeneration [62].

Peptide-based compounds that self-assemble have demonstrated significant promise for multimodal therapy, since they can adapt to changes that occur throughout complex wound healing processes [63]. Peptides that have a certain arrangement may be created by encoding peptide sequences. These peptides can form several types of structures at a microscopic level, such as spheres, vesicles, micelles, nanofibers, and nanotubes [64,65,66]. These small structures enhance the capabilities of self-assembling peptides. Some examples of these structures include nanospheres that can enclose and transport pharmaceuticals, and nanofibers that can trap liquid solutions and form hydrogels [62]. Moreover, the peptide-based fibrous network is structurally and chemically similar to fibrin in the extracellular matrix (ECM), which enables the restoration of biological functions and the repair of damaged tissues. Peptide-based hydrogels have potential as functional biomaterials due to the rational integration of functional motifs into self-assembling peptide building blocks [67].

Besides the above considerations, the design of peptides for hydrogels that are intended to promote chronic wound healing necessitates the implementation of selective enzymatic degradation. An abnormal enzymatic environment, particularly with elevated levels of matrix metalloproteinases (MMPs), is a defining characteristic of chronic lesions. If improperly regulated, MMPs, which are enzymes that degrade a variety of extracellular matrix (ECM) components, can impede the healing process design that enables the repair of wounds [68]. Peptides with a particular cleavage site for MMPs can be added to the hydrogel, enabling the hydrogel to undergo controlled degradation as the wound environment evolves. The ability of the hydrogel to respond promptly contributes to preserving its structural stability while gently dispensing its contents, as required for the healing process. Utilizing selective enzymatic degradation is a crucial approach in formulating peptides for hydrogels intended for the purpose of treating chronic wounds. Chronic wounds are distinguished by an atypical enzymatic milieu, namely with heightened concentrations of matrix metalloproteinases (MMPs) [69]. MMPs, or matrix metalloproteinases, are a group of enzymes that break down different parts of the extracellular matrix (ECM). If not well controlled, MMPs can impede the healing process. 

In contrast to other wound dressings, peptide-based hydrogels can be controlled to self-assemble under the influence of temperature, pH, ions, and enzymes, resulting in a more precise fit around the margins of incisions. In addition, the hydrogels may be prepared in advance containing cells and functional molecules, and the components of the hydrogels can be altered to accomplish various functionalities that enhance the process of wound healing [65]. As a result, the utilization of peptide-based hydrogel properties can simplify the process of obtaining a molecular design that promotes wound healing.

### 2.2. Self-Assembled Peptide Hydrogels

Functional patterns within peptides are essential in improving the therapeutic effectiveness of peptide-based hydrogels in the process of chronic wound healing. These patterns may be designed to engage with certain cellular elements, regulate the processes of wound healing, and tackle the distinct difficulties associated with chronic wounds. In contrast, hydrogels for dermal tissue repair offer a substrate for the migration and proliferation of cells to restore the damaged or lost tissue. Self-assembled peptide hydrogels, in particular, have gained significant attention due to their ability to form highly ordered and biocompatible structures. These hydrogels can mimic the extracellular matrix and provide a favorable environment for cell interactions, leading to improved tissue regeneration.

Nowadays, the phenomenon of self-assembly in biological systems has garnered significant interest due to its ability to create functioning supramolecular structures from individual macromolecules. Peptide hydrogels are a highly important type of biomaterial that have exceptional self-assembling properties [70,71]. Peptide hydrogels are a type of soft materials that utilize amino acids and peptides as building blocks. These materials have the ability to effectively capture water or fluids inside their molecular structure and transform into a hydrogel at the nanoscale level under physiological circumstances [72,73]. The molecular interactions that are responsible for the formation of these systems are primarily non-covalent, including hydrogen bonding, hydrophobic interactions, aromatic π–π stacking, and electrostatic interactions [74,75]. Heating–cooling and pH adjustment are the most common methods used to produce this type of hydrogel. Additionally, the addition of a suitable salt to the peptide solutions at a high pH is a common practice.

Furthermore, self-assembled peptide hydrogels have the ability to respond to various stimuli such as changes in pH, temperature, mechanical forces, ionic strength, and biological fluids, but the responsiveness is not universal. The ability to respond to changes in its surroundings mostly depends on the specific peptide sequence and the conditions under which self-assembly occurs. Also, they display a wide variety of sol–gel transitions, such as thixotropic gel formation. This suggests that some peptide hydrogels have thixotropic characteristics which enable them to change from a sol to a gel state when shear stress is applied; however, not all of these materials have this feature. Only certain formulations exhibit thixotropy due to their distinct structural characteristics and intermolecular interactions.

Additionally, these hydrogels have the potential to encapsulate medicinal molecules with varying characteristics through physical or chemical bonding [76,77]. The features mentioned are greatly influenced by the molecular structure of the main peptides, including β-sheets, α-helices, coiled secondary structures, and intermolecular interactions. Peptide hydrogels may be engineered with various configurations of amino acids to exhibit sensitivity to diverse stimuli. This trigger enables precise manipulation of the gelation process in terms of both time and space, hence expanding its range of potential uses.

### 2.3. Peptide-Based Hydrogels for Wound Healing

Wound healing is a complex process that involves many different cellular and molecular stages. The primary emphasis of fundamental research is on antibacterial qualities and cost-effective materials due to specific situations such as bacterial infection, renal illness, ischemia, diabetes, and local hypoxia. These factors contribute to the development of complicated wounds that require a longer healing period and pose a life-threatening risk [78]. Chronic wounds release proteolytic enzymes, including elastase, MMP, plasmin, as well as reactive oxygen and nitrogen species (ROS and RNS) [79]. These disrupt the equilibrium between the degradation and maturation processes and alter the oxidant/antioxidant state in non-healing cells. Table 1 shows the different types of peptides with their role in wound healing.

Previous research has established that reactive oxygen species (ROS) can act as a mitochondrial membrane potential (MMP) inducer, leading to necrosis and irreversible harm in damaged tissues. A recent study suggested that a reduction in ROS/RNS and elastase levels can facilitate the healing of chronic wounds by promoting the normal wound healing process [78]. They effectively discovered short peptides, namely Pep4 (KRCCPDTCGIKCL) and Pep4M (KRMMPDTMGIKML), derived from the antibacterial region of a naturally occurring elastase inhibitor [78]. These peptides have practical uses in wound dressing. The key criteria for selecting an ideal wound dressing include creating a moist environment, avoiding secondary infections, absorbing wound exudate, minimizing wound necrosis, keeping the wound from drying up, and facilitating the transport and activation of growth factors [80].

There is an abundance of reports that have developed and characterized hydrogel-based platforms for swelling, in situ gelling, small molecule delivery, and hydrophilicity. The risk of bacterial infections is reduced by the expansion capability, which prevents the formation of fluid-filled cavities. In the same way, in situ gelling can establish a crosslinked network that serves as a platform for the regulation of the delivery of small molecules (such as pharmaceuticals or growth factors) and the complete closure of the incision [79]. Antimicrobial agents, including antibiotics, AgNPs, and antimicrobial peptides (AMPs), have garnered increased attention due to their potent inhibition activity against a wide range of microbes, as a result of the trend toward the development of restorative materials with antimicrobial properties [81]. As an example, AMPs can promote the contraction capacity of fibroblasts using SR-0379 (a synthetic peptide) by inducing fibroblast-to-myofibroblast differentiation as well as enhancing the α-smooth muscle actin expression by fibroblasts such KSL-W to enhance wound healing [82].

Over the past ten years, there has been a growing fascination with the use of self-assembled peptide hydrogels as a viable substitute for conventional scaffolds in the field of regenerative medicine. The nanofibers grow spontaneously and then create a scaffold-like structure that connects tissues. Peptide self-assembly can be utilized as regenerative agents for soft tissues such as blood vessels and skin by promoting the formation of new blood vessels through angiogenesis [83]. Nanoscale peptide fibers facilitate a direct connection between the peptide scaffold, extracellular matrix (ECM), and injured tissue on both sides of the lesion, therefore enabling cell migration into the scaffold.

One example of an ionic self-complementary peptide, the RADA16-I peptide, has garnered significant interest for its capacity to enhance the growth of brain cells and the development of synapses, as well as its potential to heal wounds and restore damaged optical pathways [79]. RADA16-I can provide several features, including the formation of a nanofiber network that resembles the extracellular matrix (ECM) and creates a favorable environment for cell growth in vivo. It can also degrade into L-amino acids that can be used by surrounding tissues, without containing any biological or chemical impurities commonly found in animal-derived materials like collagen. Additionally, it avoids tissue rejection by eliciting non-immunogenic responses [84]. Nevertheless, the acidic pH of RADA16-I may limit its potential for widespread usage due to its potential to harm cells and host tissues in 3D culture. In order to improve efficiency and address this constraint, Sun et al. introduced a durable nanofiber hydrogel consisting of IKVAV derived from laminin and the RGD sequence derived from fibronectin, all at a pH level that occurs naturally.

In addition, a prior investigation presented a smart hydrogel composed of L-lysine-containing peptides that had an inherent propensity for self-assembly and the creation of helical threads [79]. It could therefore serve as an excellent foundation to expedite the healing of burn wounds in rats by maintaining an optimal level of hydration for the partial-thickness burn wound healing process. Under these conditions, they conducted a comparison analysis of two hydrogels based on peptides and a standard-of-care product (Mepitel^®^, a polyamide net covered with silicon). The preliminary investigation indicated that autolytic debridement could initiate and complete the process of removing dead tissue to stimulate the regeneration of the outermost layer of the skin and the layer underneath without the requirement of external substances that encourage development. The study demonstrated that the ultrashort peptide hydrogels addressed a specific gap that is currently overlooked by existing therapy alternatives. To improve the healing of wounds in this scenario, regenerative qualities can be further augmented by using bioactive substances including medication, antibacterial agents, growth factors, and cytokines [85]. Peptide hydrogels are particularly emphasized as scaffolding for skin regeneration, specifically for treating deep, partial, and full-thickness burns, without causing an immune response.

Preclinical investigations have identified Connexin43 (Cx43) as a target for therapy in cutaneous wound healing, since it has the potential to expedite wound re-epithelialization. A prior investigation assessed the efficacy of ACT1, a synthetic peptide mimicking the C-terminus of Cx43 consisting of 25 amino acids, in the treatment of non-healing neuropathic diabetic foot ulcers [86]. The study focused on people with ulcers lasting for a minimum of four weeks and compared the use of ACT1 with conventional care. The study determined that the inclusion of ACT1 in a hydroxyethyl cellulose hydrogel resulted in a considerable reduction in ulcer size, faster healing of the lesion, and complete re-growth of the ulcer’s outer layer without any negative consequences or immune system reactions. Similarly, the synergy between the regenerative properties of stem cells generated from adipose tissue and the therapeutic benefits of Exendin-4, a receptor agonist for glucagon-like peptide-1, resulted in a faster decrease in wound size and improved skin restoration. The combined treatment of Ex-4 and SCs surpasses migration, invasion, and proliferation in human endothelial cells and keratinocytes [86]. These proof-of-concept studies can be employed for subsequent investigations in the wound healing process and include the stated peptides into complex materials like hydrogels.

**Table 1:** Different types of peptides and their role in wound healing.

No	Type of Peptide	Name of Peptide	Role in Wound Healing	Reference
1	AMPs	Pep4 (KRCCPDTCGIKCL) and Pep4M (KRMMPDTMGIKML)	Serve as ideal wound dressing creates a moist environment, prevents infections, absorbs excess fluid, reduces necrosis, and keeps the wound from drying out.	[80]
2	SAP	RADA16-I- peptide	Provide nanofiber network forms like the extracellular matrix (ECM), providing a good environment for cell growth.	[79,84]
3	SAP	L-lysine-containing peptides	Provide a strong foundation to speed up burn wound healing in rats by keeping the wound properly hydrated.	[79]
4	Peptide incorporated into a polymer matrix	Synthetic peptide, ACT1	Reduce the ulcer size significantly, healed faster the lesion, and the outer layer fully regrew without any negative effects or immune reactions.	[86]

## 3. Physicochemical and Mechanical Properties of Peptide-Based Hydrogels

Hydrogels have drawn a lot of attention as scaffolds for a variety of applications including tissue engineering, regenerative medicine, wound healing, drug delivery, and others due to their excellent physicochemical features and broadly adjustable mechanical properties. Peptide-based hydrogels possess several physicochemical and mechanical characteristics such as biodegradability, swelling, and suitable rheological properties. The only report on peptide-based hydrogel characterizations in this part is based on a few parameters extracted from the selected articles. An extensive description follows below to provide a prominent output, as well as important points to support the current findings. Table 2 shows the physicochemical and mechanical properties of peptide-based hydrogels.

### 3.1. Physicochemical Properties: Evaluation of Physicochemical Properties of Peptide-Based Hydrogels

The physicochemical properties of peptide-based hydrogels are vital for their effectiveness and functionality across different applications. Consequently, grasping and fine-tuning these properties is crucial to ensure that hydrogels fulfil the specific requirements of their intended uses. Every characteristic is critical for promoting the various phases of wound healing.

For instance, scanning electron microscopy analysis (SEM) offers precise pictures of the hydrogels’ surface morphology. It facilitates the visualization of the hydrogel’s network structure, porosity, and surface texture. Knowing these structural characteristics is essential for chronic wound healing, due to how surface morphology influences the hydrogel’s ability to interact with the wound in a variety of ways, such as its capacity to facilitate cell attachment, absorb wound exudate, and foster tissue regeneration. Several studies have shown that a higher concentration of peptide in the hydrogel makes the ultrastructure significantly denser. This increase in peptide concentration induces the formation of a more intricate and pronounced fibrous network within the hydrogel matrix, thereby enhancing its structural complexity. Fabrizio Gelain et al. conducted research on self-assembled peptide (SAP) hydrogels by incorporating several peptide concentrations, from 20% to 30% *w*/*v*. They found that peptides formed thicker fibers and exhibited an increased number of spindle-shaped beads as their concentration increased [87]. This finding was also corroborated by the study conducted by Chaitanya Kumar Thota et al. which showed that increasing the tripeptide N-formyl-methionyl-leucyl-phenylalanine (fMLF) hydrogel concentration led to a corresponding increase in the thickness of the fiber bundles.

Furthermore, the presence of a crosslinker also may influence the ultrastructure of a hydrogel [88]. According to a study by Huang et al., their QLK peptide hydrogel exhibited more densely packed nanofibrous entanglements and a greater continuity of self-assembling fibers following crosslinking with transglutaminase (mTG). This result indicates that the packing density of the functionalized QLK peptide can be effectively modulated with the aid of transglutaminase as a crosslinking agent. However, some studies have concluded that additional components did not impart any changes in the morphology of the hydrogel [89]. Yuan et al. investigated the impact of incorporating the hemostatic self-assembling peptide AFCP [90], while Hao et al. examined the addition of the velvet antler blood peptide (VBP). Both studies found that these components did not alter the hydrogel’s structure, which remained similar to that of the hydrogel containing peptide alone [91].

On the other hand, transmission electron microscopy (TEM) provides a better resolution perspective which enables researchers to view the internal hydrogel structure at the nanoscale. TEM proves particularly helpful in observing hydrogels in conditions where peptides self-assemble into fibrils or other nanostructures that make up the hydrogel’s structure. The mechanical characteristics of the hydrogel and its capacity to imitate the extracellular matrix (ECM) are necessary to promote tissue healing and cell proliferation in chronic wounds which are greatly dependent on these nanostructures. TEM analysis provides a high-resolution insight into the structural and morphological properties of the hydrogel. Using TEM, Huang et al. accurately measured and observed the diameters and pore size of the fibers of their produced hydrogel material, finding that they closely resembled those of natural extracellular matrix (ECM) fibers [89].

Next, as Fourier transform infrared (FTIR) analysis can precisely identify chemical compositions and functional groups required for biocompatibility, it is essential for the fabrication and optimization of biomaterials used in chronic wound healing. To maintain the structural integrity and undergo controlled biodegradation in materials like hydrogels, it aids in determining the degree of crosslinking. Also, FTIR verifies the stability and existence of bioactive substances, which are essential for accelerating the healing process and include growth factors and antibacterial agents. When it comes to the design and validation of wound healing strategies, FTIR helps with quality control by identifying impurities or residual solvents, verifying that the materials are safe and effective for clinical usage. FTIR analysis assists in identifying and analyzing the functional groups present in a mixture.

FTIR can be used to verify the effect of crosslinking in peptide hydrogels crosslinked with any crosslinker, showing prominent peaks in relevant regions to demonstrate chemical interaction between the peptide and crosslinker. A study conducted by Fabrizio Gelain et al. thoroughly analyzed crosslinked self-assembled peptides (SAPs), revealing three prominent peaks in the amide I and amide II regions at 1695 cm^−1^, 1630 cm^−1^, and 1540 cm^−1^. These distinctive absorption bands provide critical insights into the structural organization and molecular interactions within the crosslinked SAPs. The peak at 1695 cm^−1^ is indicative of β-sheet conformations, while the peaks at 1630 cm^−1^ and 1540 cm^−1^ reflect the amide II region’s contribution to the overall secondary structure and hydrogen bonding interactions. The presence and intensity of these peaks elucidate the specific molecular arrangements and crosslinking effects, thereby enhancing our understanding of the peptide’s self-assembly and structural stability in the hydrogel matrix [87].

Additionally, stretching vibrations observed in FTIR spectra were demonstrated by Chawla et al. In their study, the strong amide I peak showed that carbonyl stretching was prominent, which is important for forming β-sheet structures in self-assembling peptides. The amide II and III peaks provided key information about hydrogen bonding and the stability of the peptide’s secondary structure, which is crucial for the strength of hydrogel [92]. In contradict, some research has demonstrated that the effect of the crosslinker in FTIR spectra is relatively minor. Qin et al. studied HSN@RL-QN15 nanocomposites and revealed that they had lower wave measurements at certain absorption positions, even though hydrophobic interactions were present between HSN and RL-QN15. This may be due to interactions which alter the molecular environment, affecting the vibrational frequencies and intensities of specific absorption peaks. In this study, the FTIR spectra for ZA (zinc acetate), HSN@RL-QN15/ZA (with zinc crosslinker), and Sac (saccharide) showed no significant differences. This indicates that the introduction of zinc ions as a crosslinking agent did not notably change the spectral features, implying that zinc crosslinking does not significantly impact the detectable molecular vibrations or bonding interactions within the nanocomposites [93]. 

**Figure 3:**
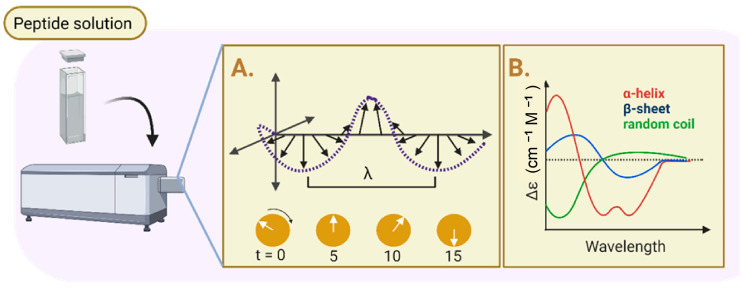
Procedure of analyzing protein secondary structure of peptide using CD. Sample is analyzed using circularly polarized light (**A**). Light interacts with peptide bond and is refracted based on associated structure (**B**). Image created by using Biorender.com.

The development of biomaterials for chronic wound healing relies greatly on circular dichroism (CD) analysis because it provides extensive insights into the secondary structures of proteins and peptides, which are essential for their bioactivity and therapeutic efficacy. In order to effectively promote cell adhesion, proliferation, and tissue regeneration, it is necessary that these molecules maintain their correct folding, structural integrity, and stability under a variety of conditions. CD supports consistent and efficient healing outcomes by enhancing biomaterial design and guaranteeing its continued efficacy in the harsh environment of chronic wounds as well as assisting in the optimization of wound healing material design by guiding hydrogels or scaffolds that mimic the extracellular matrix. Circular dichroism (CD) analysis is performed using a CD spectrometer, able to provide essential information on the secondary structure, conformational stability, and interactions of peptides in hydrogels, making it a valuable technique for designing and optimizing peptide-based hydrogel systems. Figure 3 demonstrates the procedure of analyzing the protein secondary structure of a peptide using CD.

Generally, a β-sheet secondary structure is present in all peptides including the peptide alone and peptides with crosslinkers. In the research of Huang et al., three experimental groups, the QLK peptide alone, the QLK peptide with enzymatic crosslinking, and the QLK peptide combined with LRK peptide and heparan sulfate (HS) revealed distinct characteristics indicative of β-sheet formations, suggesting that this structural motif is maintained across different conditions and modifications. The presence of β-sheet structures in these varied experimental setups highlights the stability and consistency of this secondary structure, regardless of the additional crosslinking or peptide combinations [89]. Additionally, CD analysis can characterize the β-sheet secondary structure with the presence of a positive or negative peak. As an example, Lou et al. employed CD analysis and received a result with a positive peak at 191 nm, indicative of the presence of β-sheet conformation. This is associated with specific arrangements of peptide bonds in the β-sheet structure, while a negative peak around 216 nm further confirms the β-sheet structure, as it reflects the ability of peptide to absorb circularly polarized light in a manner typical of organized β-sheet formations. These spectral features collectively suggest that the KGH peptide adopts a stable β-sheet secondary structure [94].

Another important physicochemical characterization method for peptide-based hydrogels is high-performance liquid chromatography (HPLC). HPLC is an analytical technique to separate, identify, purify, and quantify peptides from various sources. Without requiring the collection of fractions, HPLC analysis can yield all of the sample’s information. HPLC yields narrow peaks according to different chromatography columns and elution conditions, which produces fractions possessing significant activity [95]. For instance, the KGH peptide was proven by Lou et al. to have a precise molecular weight of 1719.02 Da and exhibited an impressive purity level exceeding 95%. This high purity indicates that the HPLC technique effectively separated the peptide from any contaminants or impurities [94]. Moreover, another analysis confirmed that the molecular weights of other substances were also accurate, with a purity consistently above 95% [90]. These results not only validate the precision of the HPLC method in determining molecular weights but also highlight its capability to achieve high purity levels. Overall, the findings reflect the robustness of HPLC in both characterizing the integrity of the peptides and ensuring their quality by effectively separating and quantifying them with high accuracy.

Next, biodegradation analysis demonstrates how these materials degrade and are absorbed or eliminated by the body over time, which is important for the development of biomaterials for the application of chronic wound healing. Biomaterials in chronic wounds must degrade at a controlled rate that corresponds with the healing process, as ongoing treatment is frequently needed. Ensuring the non-toxicity and lack of adverse reactions of the degradation products is crucial for patient safety, as well as to avoid aggravating wounds which are already difficult to treat. Moreover, biodegradation analysis aids in the optimization of the material’s mechanical characteristics, promising that it offers suitable support and protection during the healing process before subsequently disintegrating as the tissue regenerates.

The breakdown of materials by biological processes is referred to as biodegradation, which is primarily through the action of enzymes or microorganisms. For peptide-based hydrogels, this involves the biodegradability of peptide chains and the hydrogel matrix over time, leading to their gradual decomposition into simpler, non-toxic components. For this parameter, there are differences in biodegradability between crosslinked and non-crosslinked hydrogels. This is supported by the research of Huang et al., which was conducted on two groups of hydrogels, exhibiting significant degradation. The non-crosslinked hydrogels showed a total degradation of approximately 82%, compared to an approximate 61% degradation for the crosslinked hydrogels. The slower degradation of crosslinked hydrogels compared to non-crosslinked hydrogels is primarily due to the structural stability provided by crosslinking, which restricts enzyme and water access and requires a more complex breakdown process. Non-crosslinked hydrogels, with their less stable structure, degrade more quickly because they allow easier penetration and breakdown by environmental factors [89].

Furthermore, biodegradation analysis indicated a noticeable decline in the mass of the hydrogels over time. Hao et al. proved in their research that as the incubation period extended, there was a progressive decrease in the mass residue of both CAVBPH and CAH hydrogels. Specifically, their study showed the residual weights of the hydrogels had significantly reduced from their original 100% to 59.26% for CAVBPH and 56.85% for CAH at day 14. The slightly greater reduction observed in CAH compared to CAVBPH suggests that CAH may degrade more rapidly, which could be due to several factors, such as differences in the chemical composition, crosslinking density, or structural stability, leading to a faster loss of mass [91].

Moreover, the fabrication of biomaterials for chronic wound healing relies greatly on swelling ratio analysis, because swelling affects the ability of the biomaterial to absorb wound exudate and sustain their moist healing environment. An ideal swelling ratio serves to prevent infection and encourage tissue regeneration by allowing the substance to expand to its maximum amount of absorption without becoming overly saturated. Furthermore, the swelling behavior has an impact on the mechanical properties of the biomaterial, such its strength and flexibility. This is vital for providing the wounded region with structural support so that efficient delivery of medicinal compounds, such as growth hormones or antibiotics found within the material is facilitated by a well-balanced swelling ratio, which also helps regulate their release. As for swelling studies on peptide-based hydrogels, only a few articles have reported this parameter. The work reported by two articles show rational and similar results.

First, Hao et al. found that both CAVBPH and CAH hydrogels achieved swelling ratios exceeding 200% within an hour, highlighting their ability to rapidly absorb water [91]. This swift water uptake indicates that these hydrogels possess high hydrophilicity and a strong affinity for water, enabling them to swell quickly and significantly. Also, this finding was supported by Qin et al., in which a ZA hydrogel exhibited highly favorable swelling characteristics, achieving an impressive equilibrium swelling rate of over 1400%. Notably, this equilibrium swelling rate remained stable and unchanged even after incorporating RL-QN15 and HSN@RL-QN15 into the hydrogel [93]. This consistency in swelling behavior suggests that the addition of these components does not significantly impact the hydrogel’s capacity to absorb and retain water. The high swelling capacity ensures that the hydrogels can absorb substantial amounts of fluids, which is beneficial for applications such as wound dressings by ensuring that the material maintains its functionality and effectiveness regardless of compositional modifications.

**Table 2:** Physicochemical characterization and mechanical properties of peptide-based hydrogels.

No	Parameters	Biomaterials Used	Result	Conclusion	References
1	FTIR spectroscopy SEM analysis TEM analysis CD analysis Rheology properties	Two synthetic peptide sequence Ac FKFEFKFE-QHREDGS-NH2 (F−Q) and Ac-FKFEFKFE-GRGDS-NH2 (F−G)	FTIR: The presence of absorption peaks in amide I at 1625 cm^−1^ was higher compared to amide II at 1550 cm^−1^. SEM and TEM: Every group of peptide hydrogel has a porous, fibrous network structure. Peptide nanofibers made of F-Q, F-G, and EFK8 have the potential to create an intricate network structure with interconnections. CD: The peptide solution exhibited a positive absorption band at 191 nm and a clearly visible negative absorption band at 205 nm. Rheology: The hydrogels were all capable of withstanding the external shear force. Nevertheless, the G′ and G″ values of those four samples did not significantly differ from one another.	These peptide hydrogels have a structure similar to a network of nanofibers with great viscoelasticity. The amide I and amide II areas exhibit a high-frequency absorption band, which suggests that self-assembling nanofibers with parallel β-sheet topologies may potentially develop.	[96]
2	FTIR spectroscopy SEM analysis Biodegradability analysis Rheology properties	Lauric acid-peptide conjugate gel, LA-^L^Lys-^D^Phe-^L^Lys-NH_2_, loaded with yttrium oxide (Y_2_O_3_) nanoparticles (NLG)	FTIR: The amide I peak was detected at 1642 cm^−1^, which corresponded to stretching vibrations and was attributed to C=O. The amide II peak was identified at 543 cm^−1^, which corresponded to N−H, and the amide III peak was in the region of 1229−1301 cm^−1^, which was associated with N−H bending linked to C−N stretching. SEM: The hydrogel based on the peptides NPG and LPG; the SEM pictures showed a nanofibrous structure. Biodegradability: A high rate of degradation in LPG (~62%) over NLG (~45%) in alkaline and comparing pH 8.4 (~45%) to pH 7 (~61%), NLG breakdown less slowly. Rheology: The linear viscoelastic range of peptide conjugate gel was observed at 24.8% while 82.5% for the NP-loaded gel when the range was up to 1% and the crossover point.	Positive charges on peptide chains reduce basicity, which decreases repulsion between peptide chains and improves gel stability. The NPG peptide-based hydrogel has high storage modulus (about 4 KPa) and a notable increase in crossover points.	[92]
3	SEM analysis TEM analysis HPLC analysis Rheology properties	Amyloid fibril co-assembled peptide (AFCP)	SEM and TEM: The structure of regular peptide hydrogel is mimicked by AFCP hydrogels, which produce a net-like meshwork of fibrils that are wired nanonets. HPLC: The molecular weights are accurate, and they possess more than 95% purity. Rheology: After being crosslinked with Ca2+ and fibrinogen, the rheological stiffness of AFCP hydrogel was shown to be more than ten times enhanced.	The peptide hydrogels have a fibrous network forming inside their structure. The ACFP hydrogel has excellent mechanical properties together with distinct nanonet structures.	[90]
4	FTIR spectroscopy SEM analysis Swelling study Biodegradability analysis	Chitosan/sodium alginate/velvet antler blood peptides (CS/SA/VBPs) hydrogel (CAVBPH), CS/β-GP/SA/Ca^2+^ (CAH)	FTIR: The infrared spectrum of CAVBPH revealed clear VBP absorption bands at 1582 cm^−1^, 1404 cm^−1^, and 1111 cm^−1^, indicating that the hydrogel has been loaded with VBPs. SEM: Microscopic analysis revealed that all hydrogels had a highly porous structure, and the addition of VBPs had no effect on the porosity structure. Swelling: The swelling ratios of more than 200% were attained in less than an hour by CAVBPH and CAH due to their rapid water absorption. Biodegradability: As the incubation period increased, the mass residue of the hydrogels gradually decreased; on day 14, the residual weights of CAVBPH and CAH fell from 100% to 59.26% and 56.85%, respectively.	The hydrophilicity and interconnected porous structure of CAVBPH and CAH contribute to their good swelling ratio, making them essential for wound dressing. The diffusion coefficient of VBPs may be improved by the low molecular weight of short-chain peptides and the high porosity of CAVBPH.	[91]
5	FTIR spectroscopy SEM analysis TEM analysis Swelling study	Pro-healing peptide (RL-QN15) loaded into hollow silica nanoparticles (HSNs), zinc alginate (ZA)	FTIR: Regardless of the hydrophobic interaction between HSN and RL-QN15, the HSN@RL-QN15 nanocomposites showed lower wave measurements at both positions while the ZA, HSN@RL-QN15/ZA, and Sac spectra did not differ significantly as the crosslinking of zinc ions. SEM: The HSNs in the HSN@RL-QN15 nanocomposites consisted an outer shell and a hollow core, containing RL-QN15. The ZA hydrogels have a sponge-like surface structure and are extremely porous, with an average pore size of 18.3 μm. TEM: The transparency of the HSN@RL-QN15 nanocomposites was lower compared to the HSNs alone. Swelling: The ZA hydrogel had favorable swelling characteristics, exhibiting an equilibrium swelling rate above 1400%. The equilibrium swelling rate of ZA remained unchanged upon the addition of RL-QN15 and HSN@RL-QN15.	For microscopic view, the reduced transparency of HSN@RL-QN15 implies that RL-QN15 is present in the HSNs. The evenly sized nano-composites incorporated in the hybrid hydrogel were the cause of its rough surface. Water is absorbed by the hydrophilic groups in hydrogels, and the micropore structure permits unrestricted water flow in and out. This causes the hydrogels to swell.	[93]
6	SEM analysis TEM analysis CD analysis HPLC analysis Rheology properties	Self-assembling peptide namely KGH	SEM and TEM: After gelation, the newly formed KGH hydrogel possessed a 3D nanofiber network structure. CD: The KGH peptide exhibited a typical β-sheet secondary structure, with a positive peak at 191 nm and a negative peak about 216 nm. HPLC: The KGH peptide possessed a molecular weight of 1719.02 Da and a purity of more than 95%. Rheology: G′ was greater than G″ for the rheological property, and both G′ and G″ in the KGH solution had low values.	The KGH hydrogel has low elasticity and low viscosity, as demonstrated by its low loss and storage modulus (G′ and G″).	[94]
7	SEM analysis TEM analysis Rheology properties	Hyaluronic acid (HA), Diphenylalanine (FF)	SEM: B-FF hydrogel exhibited smooth silky fibers, while the composite hydrogels had clusters of block mass. The SEM pictures of the N-FF hydrogel revealed flaky form structures, whereas the N-FF/HA gels featured microporous structures. TEM: The fibrous networks inside composite hydrogels were physically crosslinked by entangled soft nanofibers which was similar to the peptide hydrogels alone. Rheology: N-FF hydrogels had substantially greater G′ than B-FF and P-FF, while G′ of N-FF/HA and B-FF/HA increased slightly.	The good mechanical properties were obtained for all composite hydrogels. Nevertheless, the N-FF-based hydrogels exhibited significantly better mechanical properties at low concentrations compared to others.	[97]
8	SEM analysis FTIR analysis CD analysis Rheology properties	Self-assembling peptide namely EAK16 and RADA16	SEM: Thicker fibers with a few spindle-shaped beads develop when the SAP hydrogel concentration is further increased (20–30% *w*/*v*). FTIR: The crosslinked SAPs of FT-IR spectra in the amide I and amide II regions feature three prominent peaks located at 1695, 1630, and 1540 cm^−1^. CD: The CD spectra of β-forming SAP were expanded using a 7-residue functional motif and a variable-length Gly spacer. Rheology: If SAP hydrogels were crosslinked instead of non-crosslinked, their stiffness would increase from 0.1 to 100 KP.	SEM picture of the crosslinked SAP shows lengthy, tangled nanofibers that are aligned. Both conventional and crosslinked SAPs exhibit cross-β structuration, as confirmed by the results of their FT-IR spectra.	[87]
9	SEM analysis TEM analysis CD analysis Rheology properties	Self-assembling peptide namely N-formyl-methionyl-leucyl-phenylalanine	SEM: The hydrogels in every group exhibit dense networks of bundles of nanofibers in their ultrastructure. Qualitatively, it appears that the thickness of bundle increases as the fMLF peptide concentration increases. TEM: The presence of fMLF causes the nanofibers that make up the LΔF hydrogel to become thicker. CD: The increases of fMLF content in the mixtures, there were a slight decrease in intensity of the minima at 275 nm. Rheology: LΔF alone has a G′ value of 132 kPa, while LΔF/fMLF (1:1) has a G′ value of 189 kPa which these show that both co-assembled hydrogels are classified as soft hydrogels.	Intermolecular interactions might be the factor responsible for the apparent rise in nanofiber diameter. An increase in the concentration of fMLF content does not result in any apparent secondary structural changes.	[88]
10	SEM analysis TEM analysis CD analysis Biodegradability analysis Rheology properties	Self-assembling peptide namely RADA16-GGQQLK (QLK) and RADA16-GGLRKKLGKA (LRK)	SEM: More densely packed nanofibrous entanglements and more continuous self-assembling fibers appeared with the QLK peptide post mTG crosslinking. TEM: Similar to natural ECM fibers, the interwoven nanofibers had diameters of of 10–20 nm and pore sizes of 10–200 nm. CD: The β-sheet secondary structure is exhibited by three experimental groups: QLK peptide, QLK peptide with enzymatic crosslinking and QLK peptide combined with LRK peptide and HS. Biodegradability: After 35 days, both groups displayed degardation, with a total degradation of roughly 61% (crosslinked) and 82% (non-crosslinked). Rheology: QLK hydrogel showed a rise in its storage modulus (G′) from 1000 Pa to 5000 Pa, whereas QLK/LRK hydrogel had an increase in G′ from 400 Pa to 2500 Pa.	The mechanical properties of hydrogels were likewise impacted by the transglutaminase: as the concentration of mTG increased, the storage modulus (G′) of hydrogel increased as well. Comparing the crosslinking group to the non-crosslinking group, the crosslinking group’s hydrogel disintegrate rate was slower.	[89]

### 3.2. Mechanical Test: Evaluation of Mechanical Properties of Peptide-Based Hydrogels

Mechanical properties are crucial for determining the handling and durability of hydrogels. In order to assess these characteristics, a very efficient approach entails the utilization of a specialized instrument known as a rheometer which was specifically built to quantify the viscoelastic properties of materials including hydrogels. Moreover, comprehending the correlation between the chemical composition and macroscopic characteristics of hydrogels is facilitated by an understanding of their rheology [98]. Studies on the flow and deformation of materials are essential to 3D bioprinting, especially for the development and applications of bioinks. For the bioink to be extruded smoothly through the printer nozzle without clogging, as well as to retain its shape once deposited, the viscosity of the bioink must be carefully controlled.

**Figure 4:**
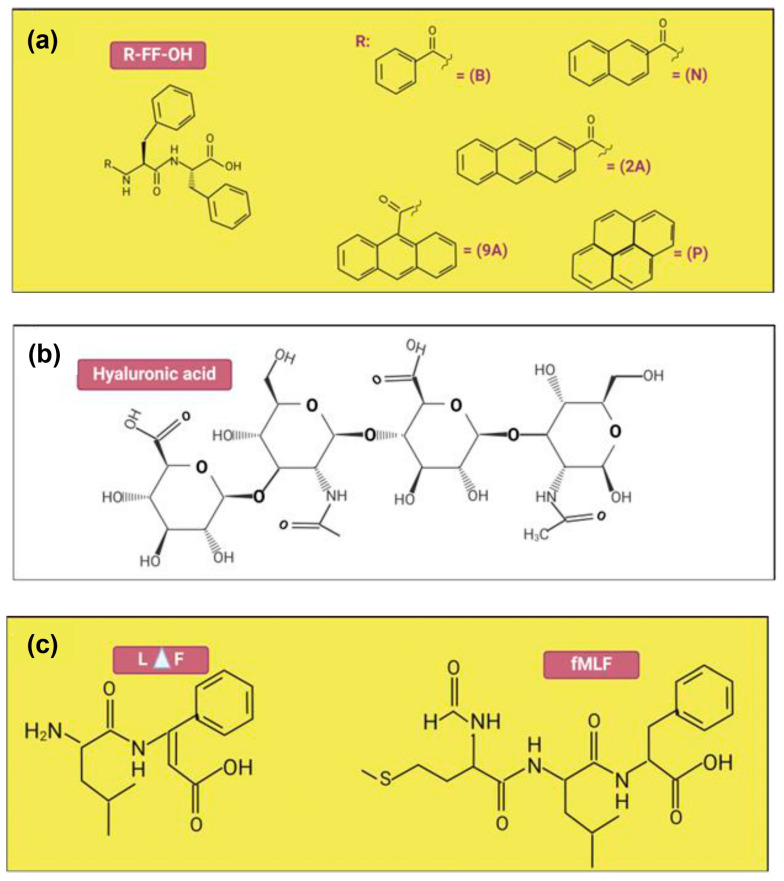
Chemical structures of (**a**) RFFOH with different diphenylalanine (FF) derivatives modified by a few aromatic moieties such as benzene (B), naphthalene (N), pyrene (P), 2-anthracene (2A), and 9-anthrene (9A), (**b**) hyaluronic acid, and (**c**) line structure of L-delta phenylalanine and N-formyl-methionyl-leucyl-phenylalanine. Image created by using Biorender.com.

The instruments to measure and modify viscosity to get an appropriate balance are provided by rheology. Also, rheological factors also affect layer fidelity and extrusion consistency. Determining the consistency of extruded bioinks is crucial, as it impacts the printed structure’s resolution and quality. On the other hand, layer fidelity can help the bioink retain its shape and support subsequent layers without collapsing or spreading. When bioinks are properly characterized, their flow may be regulated to facilitate high-precision printing. This also aids in the design of stable layer formation, which is necessary for the construction of complicated 3D structures. Additionally, by comprehending the rheological properties of bioinks, we can modify the gelation and crosslinking processes as two essential processes in stabilizing the printed structures. Rheological measurements, as reported by Fabrizio Gelain et al., revealed that the rheology of a fluorenyl methoxycarbonyl (Fmoc)-tripeptide hydrogel increased with the presence of crosslinker in the group of hydrogels [87]. Specifically, the stiffness of the hydrogels rose from 0.1 kilopascals (KP) to 100 kilopascals (KP) upon crosslinking (natural crosslinker; genipin) compared to the non-crosslinked group. This substantial increase in stiffness indicates that the crosslinking process enhanced the mechanical strength and rigidity of the hydrogels, making them more robust and resistant to deformation. In contrast, non-crosslinked hydrogels exhibited a much lower stiffness due to the absence of the structural reinforcement provided by crosslinks. Other than that, Yuan et al. demonstrated that after crosslinking with Ca^2+^ and fibrinogen, the stiffness of their AFCP hydrogel was increased by more than tenfold. The incorporation of Ca^2+^ and fibrinogen effectively reinforced the hydrogel network, leading to improved structural integrity, creating a more rigid and durable hydrogel [90]. Nevertheless, some findings have reported that the addition of biomaterials or compounds into peptide-based hydrogels does not always lead to significant changes in rheological properties. For instance, rheology analysis conducted by Wang et al. showed that naphthalene-diphenylalanine (N-FF) hydrogels had a significantly higher storage modulus (G′) compared to B-FF and P-FF hydrogels, indicating that N-FF hydrogels exhibit superior mechanical strength and stiffness [99]. The chemical structure of RFFOH with different diphenylalanine (FF) derivatives was illustrated in Figure 4. 

Conversely, the incorporation of hyaluronic acid (HA) into N-FF and B-FF hydrogels resulted in only a slight increase in their G′ values, suggesting that HA has a minimal impact on their rheological characteristics. Despite these observations, all the hydrogels demonstrated generally favorable rheological properties, with no significant overall differences observed when HA was added. This analysis highlights the robust mechanical performance of N-FF hydrogels while demonstrating that HA’s effect on enhancing rheological properties is relatively modest. However, Chaitanya Kumar Thota et al. described their peptide-based hydrogels as soft hydrogels, based on the measured storage modulus (G′) values. The G′ value for the LΔF hydrogel alone was 132 kPa, and for the co-assembled LΔF/fMLF hydrogel, it was 189 kPa. Despite the increase in G′ with the addition of fMLF, both hydrogels were classified as soft due to their relatively low G′ values [88]. In rheology analysis, soft hydrogels are characterized by lower G′ values, indicating that they are flexible and can undergo considerable deformation under applied stress.

**Figure 5:**
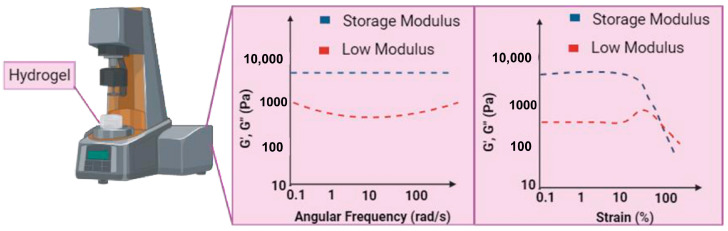
Presents the analysis of rheological property of peptide-based hydrogel using rheometer. Image created by using Biorender.com.

The increase in stiffness with the addition of fMLF, while notable, does not elevate the overall rigidity to a level that would change their classification from soft to more rigid. Therefore, both hydrogels retained their classification as soft hydrogels, reflecting their inherent mechanical properties and flexibility. Figure 5 demonstrates the procedure of analyzing mechanical strength of peptide-based hydrogel using CD. 

## 4. Biocompatibility and Cell Interaction of Peptide-Based Hydrogels Against Cells

In the field of biomaterials, biocompatibility testing is crucial. It ought to be studied to understand the capabilities and function of a fabricated scaffold, particularly in tissue engineering and regenerative medicine [100]. Besides this, biocompatibility is also known as one of the most important things to consider when employing a biomaterial for wound healing applications. Biocompatible biomaterials play a critical role in the context of chronic wounds by effectively supporting tissue regeneration and recovery while avoiding undesirable outcomes including inflammation, infection, or immunological responses. These biomaterials need to maintain cell viability and promote the formation of new tissue without releasing any toxic compounds that can worsen the wound or cause systemic toxicity.

Likewise, biocompatible materials have been designed to blend in smoothly with the body’s normal functions, creating a stable environment that promotes healing and lowers the possibility of long-term problems. It is important to ensure that scaffolds, wound dressings, and other therapeutic materials are biocompatible, meaning that they comply with regulations but also help to keep chronic wounds from getting worse, leading to better results and promoting long-term healing. Numerous studies have supported the significance of biocompatibility in biomaterials, including a study by Wang et al. on the development of hydrogels with peptide sequences of Phe-Glu-Lys-Phe (Fmoc-FEKF) and fluorene methoxycarbonyl groups, which demonstrated 96% of fibroblast cells indicating high cell viability and non-toxicity [99]. 

Another study describes the creation of multifunctional injectable hydrogels from N-(2-hydroxypropyl)-3-trimethylammonium chitosan chlorides (HTCC), a biocompatible antibacterial polymer, and demonstrates how these hydrogels can be used to treat wounds in an effective and secure way. Upon subcutaneous implantation in mice, the gel exhibited minimal toxicity to human red blood cells (about 2–3% hemolysis) and no inflammation of the surrounding tissue, indicating its efficacy as a reliable and secure antibacterial sealant [101]. 

According to various studies, peptide-based hydrogels have demonstrated promising biocompatibility and cellular interactions. A study by Kim et al. highlighted that the presence of substance P in the peptide hydrogel could stimulate fibroblast proliferation and angiogenesis and enhance cell recruitment for treating ischemic diseases [102]. Stern and Cui et al. evaluated the cellular interactions of self-complementary peptide hydrogels such as RAD16 with active motifs like IKVAV and RGD, which functioned to increase cell differentiation, adhesion, and migration [103]. Furthermore, another recent study by Rijal and Narmoneva et al. reported a RAD16 peptide nanofiber with dual functional motifs, which allowed for increased migration and proliferation of keratinocytes and fibroblasts [104]. Beneficial cellular interactions between a peptide-based hydrogel and cells were evaluated by Carrejo et al., who showed that cells exhibited fibroblast morphology at day 7 and continued to grow and proliferate within the multidomain peptide (MDP) hydrogel at day 10. In this study, the researchers hypothesized that the MDP hydrogel was able to support cell growth and proliferation in vitro, as well as promote cellular infiltration and angiogenesis, as demonstrated in a subcutaneous in vivo model [105]. Kang et al. also developed another type of hydrogel from MDP consisting of the 16-amino acid sequence K2(SL)6K2, which self-assembled into a nanofibrous hydrogel, showing excellent biocompatibility [106]. 

In vitro, MDP hydrogels facilitated the cell growth of fibroblasts and promoted migration and showed a higher level of proliferation for human umbilical vein endothelial cells (HUVECs). López-Gutierrez et al. characterized a GHK peptide, which significantly increased the expression of basic fibroblast growth factor and endothelial growth factor, aiding tissue repair through vasodilation, anticoagulation, and angiogenesis in an in vivo study [107]. Biocompatibility tests were conducted using a CCK-8 kit, which demonstrated that the F−Q/F−G hydrogel had the highest cell viability on days 4 and 7. The results of the live/dead test also showed a substantial amount of green fluorescence with minimal red fluorescence, indicating a high rate of cell viability [96]. Another study demonstrated a water-soluble peptide, QHREDGS, which could promote the survival, adhesion, and collective migration of various cells including keratinocytes to [108]. 

Cytotoxicity is an important index for the practical application of hydrogel dressings. Hao et al. conducted a cell viability test for CAH and CAVBPH groups of a test hydrogel. resulting in 81.29% and 86.74% cell viability, respectively, which is considered as no toxicity and sufficient biosafety [91]. Also, the effect of a peptide nanofiber membrane was evaluated on the proliferation of HaCat and U937 cells an in vitro cytotoxicity test, and the viability results displayed no significant cytotoxicity [109]. Lou et al. also reported that a KGH hydrogel had no cytotoxicity in a cytotoxicity assay for HaCat cells, indicating its ideal biocompatibility for skin cell growth [94]. Lastly, one study by Kim et al. reported that a composite peptide hydrogel showed no cytotoxicity and slightly promoted fibroblast differentiation [110]. Table 3 displays the cell interactions of peptide-based hydrogels for in vitro and in vivo studies.

### Peptide-Based Hydrogels in In Vivo Model Studies

Preclinical research using animal models has demonstrated the great efficacy of peptide-based hydrogels in promoting chronic wound healing. These hydrogels have excellent biocompatibility and have demonstrated fast wound closure, tissue regeneration, and inflammation reduction. The evidence emphasizes how promising peptide-based hydrogels are as novel therapeutic agents for the treatment of chronic wounds. However, further research and clinical trials are required to confirm these encouraging findings. Preclinical trials are required to validate the effectiveness, safety, and long-term advantages of these hydrogels, guaranteeing their successful integration into clinical practice to enhance patient outcomes.

This finding is supported by Kim et al. who demonstrated that a peptide hydrogel derived from a laminin-based dodecapeptide effectively served as a wound dressing in a rat wound healing model, leading to substantial improvements in wound closure, re-epithelialization, and granulation tissue formation in the wounds of diabetic rats. This composite peptide hydrogel was shown to be non-cytotoxic, facilitated well-organized collagen deposition, and reduced inflammation and scar formation, achieving nearly complete wound closure [110]. Moreover, Kang et al. evaluated two types of peptide hydrogel in an in vivo study; multidomain peptide hydrogel (MDP) and self-assembling peptide (SAP). Their findings indicated that the MDP hydrogel enhanced granulation tissue formation and re-epithelialization in diabetic mice with full-thickness wounds and accelerated wound closure compared to treatments with buffer only or Intrasite [106].

**Figure 6:**
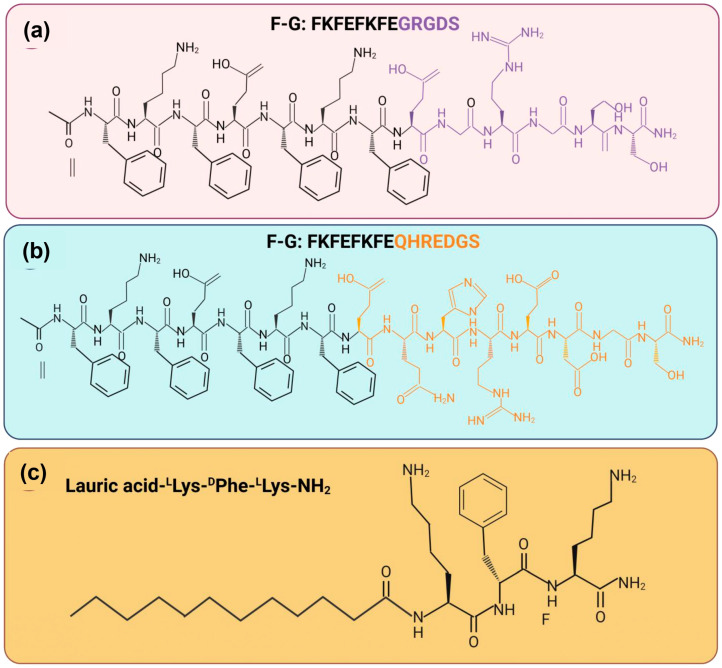
Chemical structure of peptide sequences consists of phenylalanine (F), lysine (K) and glutamic acid with additional amino acids in (**a**) glycine (G), arginine (R), aspartic acid (D) and serine (S) while in (**b**) glutamine (Q), histidine (H), arginine (R), glutamic acid (E), and serine (S); (**c**) self-assembly of lauric acid-peptide. Image created by using Biorender.com.

Likewise, in another study SAP hydrogel treatment resulted in faster closure and thicker epidermis and dermis compared to other control groups. Notably, wounds treated with Intrasite healed more slowly than those treated with MDP, which achieved faster closure [103]. In another study conducted by Gao et al., an injectable hydrogel containing a multi-domain peptide (MDP) was utilized to cure full-thickness dermal wounds in diabetic mice model, without any need for external growth factors or cells. It was discovered that the hydrogel could stimulate fibroblast proliferation and growth as well [111]. Additionally, Mandla et al. conducted preclinical studies in a diabetic mouse model by using Q-Peptide hydrogel as a treatment to cure the wound. The findings for the peptide hydrogel demonstrated that it enhanced the re-epithelialization of the wounds by inducing fast keratinocyte migration through interactions with β1-integrin [112]. Moreover, an in vivo cytotoxicity assay was carried out by Li et al. who used HE staining to observe the internal organs of mice treated with peptide hydrogels [96]. All of the internal organs of the mice such as the lung, heart, kidney, spleen, and liver were maintained in a normal structure, indicating that the peptide hydrogels were not toxic to the mice. Also, mouse back skin wound healing demonstrated that F-Q/F-G, F-Q, and F-G (chemical structure illustrated in Figure 6) peptide hydrogel treatment greatly accelerated healing of the wound, with the wound size decreasing progressively on days 3 and 7, and with all groups obtaining basic wound closure by day 14. New hair follicles which closely resembled normal skin were also visible at the healing site. Another study by Huang et al. injected a single peptide solution directly into bleeding sites in rat livers to create an in situ hydrogel [89]. This demonstrated that the optically transparent hydrogel easily conformed to lesion cavities and trapped ECM proteins and red blood cells in place, indicating the potential uses of SAP hydrogels in oozing wounds or even minimally invasive surgical procedures. The treatment ceased bleeding without the need for chemical crosslinked adhesives, cauterization, or even vasoconstriction. Moreover, another study designed a co-assembled hydrogel with a peptide and β-cyclodextrin, which exhibited antibacterial activity against Gram-positive bacteria; the hydrogel showed in vivo wound healing efficacy based on histopathological and biochemical evaluations [75]. Furthermore, a different study reported the fabrication of an antioxidant supramolecular hydrogel based on a feruloyl-modified peptide and glycol chitosan, which was crosslinked using laccase; the hydrogel was able to accelerate the regeneration of mature epithelium and connective tissues in a full-thickness skin defect model [113].

## 5. Applications of Peptide-Based Hydrogels in Chronic Wound Healing

**Figure 7:**
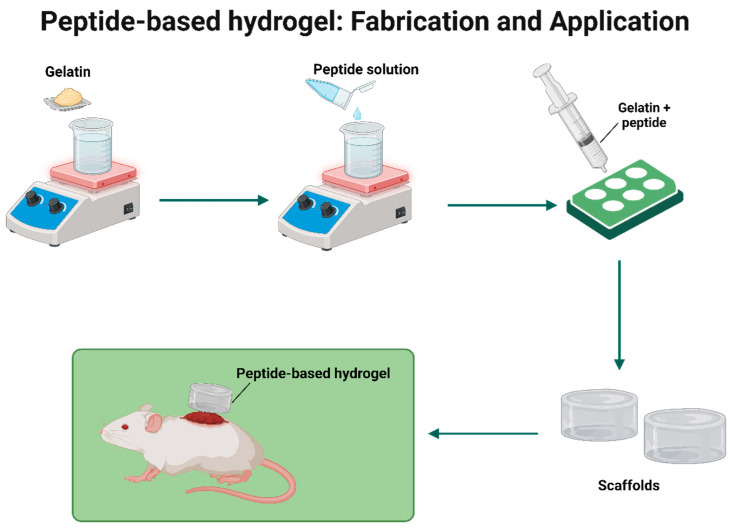
Fabrication of peptide-based hydrogel and the application of hydrogel in in vivo studies by using diabetic rat model. Image created by using Biorender.com.

Chronic wounds convey a significant healthcare issue because of their intricate healing mechanism and numerous obstacles that hinder the healing process, including infections, inflammation that is not under control, malfunctioning cells, deficient angiogenesis, and increased protease activity. Biomaterials for wound healing must address additional complexities related to wound chronicity in addition to promoting wound healing by acting as a matrix since chronic wounds are accompanied by a range of complexities. Thus, it is necessary to discuss the application of peptide-based hydrogels in wound healing, specifically in chronic wound healing. Generally, Figure 7 shows the fabrication of peptide-based hydrogels and the application of such hydrogels in in vivo studies.

Numerous investigations have been carried out to validate the efficacy of peptide-based hydrogels in the treatment of chronic wounds. For example, Balaji et al. have discovered that peptide nanofibers (NFs) facilitate the development of a stable in situ tissue-engineered provisional matrix (ISTEPM), which enhances the local diabetic wound microenvironment, encourages granulation tissue deposition and neovascularization, reduces inflammation, and, with the combined effects of these processes, improves diabetic wound healing [114]. As a way to improve wound morphology, speed up wound closure, and enhance the outcome of wound repair, the results imply that ISTEPM formation in NF-treated wounds may attenuate inflammation and alleviate diabetes-associated deficiencies in vascular cell infiltration and wound neovascularization at early stages of wound healing. This might therefore be a useful strategy for future research into novel treatments for chronic diabetic ulcers in human patients.

In a different study by Carrejo et al., an MDP hydrogel dramatically sped up wound healing when applied to full-thickness dermal wounds in genetically diabetic mice in comparison to a hydrogel that is currently in use with a control buffer [105]. After receiving treatment with the MDP hydrogel, the lesion healed in 14 days and thick granulation tissue with extensive innervation, vascularization, and hair follicle regeneration developed. This implies that treating patients with diabetic wounds with an MDP hydrogel would be a desirable option. Furthermore, two topical treatments involving the reformulation of valsartan into a self-assembling filament hydrogel by Nidadavolu et al. enabled the treatment to release over a prolonged period of time and functioned as a scaffold in the wound bed of diabetic rats [115]. In comparison to one wound that healed in the placebo group on day 23, peptide-based hydrogel treatments on full-thickness wounds in Zucker Diabetic Fatty (ZDF) rats produced faster rates of wound closure, and all val-filament treated wounds were completely closed. Following the inflammatory phase of wound healing, the administration of a val-filament hydrogel led to accelerated wound healing, increased density of hair follicles, altered cell adhesion pathways, downregulated expression of Smad as a mediator of the Tgf-β signaling pathway, and improved mitochondrial energetics. Moreover, Gao et al. observed a high acceleration of collagen deposition and angiogenesis by applying self-assembling peptide hydrogels with substance P for injection treatment of a diabetic wound [111]. Similarly, Kim et al. demonstrated a progressive reduction in wound area (predominantly filled with regenerated tissue) after the treatment of groups R+SP and R+R-SP with SAP and substance P. Three weeks following treatment, substance P had accelerated wound healing [102]. Plus, by attracting MSCs, which are engaged in the earliest stages of wound healing, these data suggest that substance P not only speeds up wound healing but also reduces the overall size of the wound.

Also, a different nanofiber hydrogel (multidomain peptide K2(SL)6K2) created with the same self-assembly mechanism was shown to speed up the creation of granulation tissue, re-epithelialization, vascularization, and innervation in diabetic wounds [106]. Moreover, Mandla et al. demonstrated that Q-peptide treatment for diabetic mice and rats with chronic wounds is highly successful, leading to progressive wound closure and not just wound repair but also whole body tissue restoration after injuries [112]. Last but not least, research by Wan et al. indicated that applying a scorpion peptide gel to rats with ulcers caused the ulcers to heal more quickly than the epidermal growth factor group did, particularly from the second week to the point at which the ulcer fully healed following ulcer induction [116]. This may have occurred because the inflammatory factors and microbial growth on the surface of diabetic ulcers were more effectively inhibited by the scorpion peptide gel.

## 6. Challenges, Limitations, and Future Perspective

Peptide-based hydrogels are becoming more and more popular in applications involving chronic wound healing because of their capacity to stimulate tissue regeneration, their biocompatibility, and adjustable characteristics. However, before they can be widely used in therapeutic settings, a number of issues and restrictions must be resolved. Peptide-based hydrogels have the potential to break down prematurely before the wound has fully healed due to their susceptibility to enzymatic degradation in vivo, which can affect both the stability and pace of breakdown. Controlling the rate of deterioration to align with the wound healing process poses a challenge and may impact the treatment’s effectiveness. Moreover, the adoption of peptide-based materials for clinical usage is severely hampered by their cost and scalability. These materials cannot be produced on a large scale because their synthesis is frequently costly and requires intricate procedures. Their high cost and complexity prevent peptide-based hydrogels from being widely used in therapeutic settings. It is imperative to develop affordable production procedures that can be scaled up without sacrificing the hydrogels’ quality or usefulness in order to make these sophisticated materials commercially feasible.

In biomedical applications, controlled drug release from peptide-based hydrogels is a significant difficulty. The intrinsic complexity of these materials makes it challenging to provide precise control over the release of medicines. The efficacy of therapies can be greatly impacted by the erratic release patterns frequently linked to peptide-based hydrogels, which makes it more difficult to guarantee that the right dosage is administered at the right time. This volatility makes dosage control more difficult, which may result in less-than-ideal therapeutic results and higher patient hazards. Resolving this issue is crucial to improving the dependability and efficacy of drug delivery systems.

In order to overcome significant shortcomings in the present materials, future peptide-based hydrogel research could investigate the creation of peptide sequences that provide both mechanical strength and controlled degradation. Through optimization of these sequences, hydrogels that meet therapeutic objectives and preserve structural integrity at a regulated rate could be produced. Hydrogels could be made more resilient and adaptable by adding crosslinking techniques or hybridizing peptides with other biocompatible materials to improve their qualities. The performance and suitability of peptide-based materials in biomedical applications would be greatly enhanced by these developments. Besides the above, the peptide-based hydrogels with multifunctionality have the potential to transform treatment approaches by combining several therapeutic ingredients onto one platform. Antimicrobial peptides and growth factors are examples of drugs that can be embedded within hydrogels to treat numerous aspects of wound healing at the same time. Since the hydrogels can not only help the healing process generally and prevent infections but also encourage tissue regeneration, this technique enables a more thorough and successful course of treatment. The results of wound care and other medical applications may be considerably improved by the capacity to administer these various therapeutic substances in a regulated and cooperative manner.

The currently developing field of regenerative medicine uses cutting-edge treatments to replace or repair damaged tissues and organs. Combining peptide-based hydrogels with stem cells or other regenerative therapies is a potential strategy in this field. Peptide-based hydrogels facilitate tissue healing and cell proliferation by offering a supporting matrix that resembles the extracellular milieu. These hydrogels have a far higher potential for chronic wound healing when paired with stem cells or other regenerative therapies. This synergistic combination not only promotes more substantial tissue regeneration but also speeds up the healing process, raising the possibility of more successful therapies for chronic wounds. The cost and accessibility of cutting-edge medical materials like peptide hydrogels depend heavily on sustainable production. Recombinant peptide technology and green chemistry approaches are two examples of cost-effective and sustainable production methods that can be developed to drastically lower the overall cost of producing these hydrogels. Green chemistry focusses on ecologically friendly procedures that reduce waste and energy usage, while recombinant peptide technology uses genetically modified organisms to generate peptides efficiently. When combined, these creative methods may reduce production costs and increase the accessibility of peptide hydrogels for a range of uses, such as regenerative medicine and healthcare.

## 7. Conclusions

**Figure 8:**
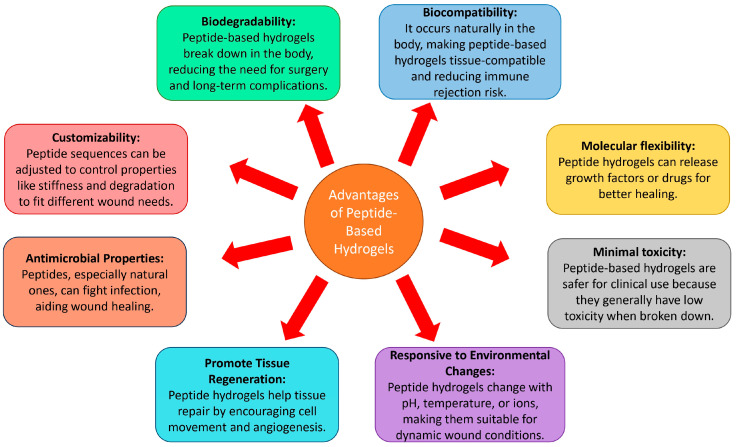
The advantages of the peptide-based hydrogels in wound healing application.

The review provides a comprehensive overview of the key findings in the field of wound healing, shedding light on the intricate mechanisms that govern the healing process. It draws attention to the critical functions that growth factors like PDGF, VEGF, and TGF-β play in controlling cell migration, proliferation, and differentiation during the healing process. The review also emphasizes how important the ECM is for supplying the structural support and signaling molecules needed for tissue restoration. The potential of cutting-edge treatments is also examined; stem cell-based therapies, gene therapy, and bioengineered scaffolds are among the novel methods that appear to have promise for improving outcomes and hastening the healing of wounds, especially those that are chronic and difficult to cure. Wide-ranging consequences for future studies point to a paradigm shift in favor of more individualized and focused therapy approaches. Subsequent investigations may concentrate on enhancing the transportation and effectiveness of growth factors, creating biomaterials that imitate extracellular matrix, and delving deeper into the function of immune regulation in wound healing. Next-generation wound care products that are customized to each patient’s unique needs may result from the combination of cutting-edge technologies like nanotechnology and 3D bioprinting. These developments have the potential to greatly improve patient care by expanding our knowledge of wound healing and influencing clinical procedures. The field’s ongoing evolution presents an increasing opportunity to create innovative approaches that tackle the many problems associated with wound healing, which should ultimately improve therapeutic outcomes and lower healthcare costs. Figure 8 shows the summarization of advantages of peptide-based hydrogels.

## Figures and Tables

**Table 3:** Cell interaction of peptide-based hydrogel for in vitro and in vivo studies.

No	Type of Peptide Hydrogel	In Vitro	In Vivo	Ref.
1	Self-assembling peptide hydrogel (SAP)	The Ac-FKFEFKFE-QHREDGS-NH2 (F−Q)/Ac-FKFEFKFE-GRGDS-NH2 (F−G) hydrogels exhibit excellence biocompatibility as determined by CCK-8 kit, resulting in an increased number of endothelial cells which led to highest cell viability at day 4 and 7.	No cytotoxicity effects in internal organs of mice that received peptide hydrogel. Greater cell proliferation and re-epithelialization were observed in the F-Q/F-G group, leading to nearly full closure of wound as well as appearance of new hair follicles at healing site.	[96]
2	Functionalized peptide hydrogel	CAH and CAVBPH shows 81.29% and 86.74% cell vitality, respectively, when the concentration of peptide was 5000 μg/mL. More than 75% cell viability was obtained, indicates that these peptide hydrogels have good biosafety and minimum toxicity.	On day 15, the AVBPH group had the highest rate of wound healing, at 96.55%, indicating the best treatment effectiveness. Pathological staining demonstrated CAVBPH’s positive impact on collagen deposition, fibroblast proliferation, angiogenesis, re-epithelialization, and inflammation regulation.	[91]
3	Crosslinked peptide hydrogel	The effects of the hydrogels ZA, RL-QN15/ZA, and HSN@RL-QN15/ZA (500 μg/mL) may promote keratinocyte migration and proliferation of cells. the viability of cell proliferation was demonstrated by ZA and HSN@RL~QN15/ZA, with the maximum values of 132.61 ± 8.74 and 159.74 ± 15.29%, respectively.	Hematoxylin and mouse mortality were not detected when HSN@RL-QN15/ZA hydrogel was applied topically to dorsal skin wounds. Instead, angiogenesis was regulated, inflammation was decreased, and the creation of granulation tissue and re-epithelialization was expedited, leading to a quick healing response.	[93]
4	Self-assembling peptide hydrogel (SAP)	The KGH hydrogel promoted the production of growth factors and extracellular matrix (ECM) proteins in skin keratinocytes, supported the formation of cell spheroids, and produced a 3D microenvironment for skin cells.	Up to seven days following injection, the KGH hydrogel remained in the wounds and effectively accelerated wound closure by around 20% as compared to the control groups. The result was achieved by boosting angiogenesis, cell proliferation, granulation tissue development, and extracellular matrix deposition/ remodelling.	[94]
5	Self-assembling peptide hydrogel (SAP)	The R+SP group was discovered to have a greater contribution to the development of keratinocytes in wounded skin, as evidenced by the robust expression of cytokeratin 14 and involucrin positive cells in the wound after three weeks.	The wound area s with R+SP and R+R-SP groups were primarily filled with regenerated tissue, gradually shrank after receiving treatment with SAP and substance P. These findings suggest that substance P in peptide hydrogel speeds up wound healing.	[102]
6	Self-assembling peptide hydrogel (SAP)	SAP that included stromal cell-derived factor-1 (SDF1-ELP), enhanced endothelial cells migration, proliferation, and vascularization.	Greater vascular endothelial cells (CD31+ cells), quicker wound closure, and much thicker epidermis and dermis were observed when SDF1-ELP in fibrin gel was introduced to in vivo diabetic wounds in mice as opposed to free SDF1 and other control groups.	[106]
7	Multidomain peptide hydrogel (MDP)	K2 hydrogel exhibited biocompatibility, which allowed for rapid cellular infiltration to promote granulation tissue formation, angiogenesis, and hair follicle regeneration as well as act as a degradable matrix to facilitate cell infiltration including fibroblasts.	When tested as a dressing in a diabetic rat wound model, the K2 hydrogel groups demonstrated a significantly greater healing rate with almost complete closure. This composite hydrogel also showed no cytotoxicity and promoted fibroblast differentiation.	[110]

## Data Availability

The data presented in this study are available on request from the corresponding author.

## References

[1] Man E., Hoskins C. (2020). Towards advanced wound regeneration. Eur. J. Pharm. Sci..

[2] Rittié L. (2016). Cellular mechanisms of skin repair in humans and other mammals. J. Cell Commun. Signal..

[3] Xu Z., Han S., Gu Z., Wu J. (2020). Advances and Impact of Antioxidant Hydrogel in Chronic Wound Healing. Adv. Healthc. Mater..

[4] Raziyeva K., Kim Y., Zharkinbekov Z., Kassymbek K., Jimi S., Saparov A. (2021). Immunology of acute and chronic wound healing. Biomolecules.

[5] Fadilah N.I.M., Phang S.J., Kamaruzaman N., Salleh A., Zawani M., Sanyal A., Maarof M., Fauzi M.B. (2023). Antioxidant Biomaterials in Cutaneous Wound Healing and Tissue Regeneration: A Critical Review. Antioxidants.

[6] Malone-Povolny M.J., Maloney S.E., Schoenfisch M.H. (2019). Nitric Oxide Therapy for Diabetic Wound Healing. Adv. Healthc. Mater..

[7] Rodrigues M., Kosaric N., Bonham C.A., Gurtner G.C. (2019). Wound healing: A cellular perspective. Physiol. Rev..

[8] Singer A.J., Clark R.A. (1999). Cutaneous wound healing. N. Engl. J. Med..

[9] Flynn K., Mahmoud N.N., Sharifi S., Gould L.J., Mahmoudi M. (2023). Chronic Wound Healing Models. ACS Pharmacol. Transl. Sci..

[10] Andreassi A., Bilenchi R., Biagioli M., D’Aniello C. (2005). Classification and pathophysiology of skin grafts. Clin. Dermatol..

[11] Wang C., Zhang F., Lineaweaver W.C. (2020). Clinical Applications of Allograft Skin in Burn Care. Ann. Plast. Surg..

[12] Esmaeili A., Soleimani M., Rouhani M., Noorkhajavi G., Aghaei-Zarch S.M., Hasannejad-Asl B., Bagheri-Mohammadi S., Ebrahimi M., Keshel S.H. (2024). Xenograft-based skin substitutes: A critical review. J. Drug Deliv. Sci. Technol..

[13] Jaller J.A., Herskovitz I., Borda L.J., Mervis J., Darwin E., Hirt P.A., Lev-Tov H., Kirsner R.S. (2018). Evaluation of Donor Site Pain after Fractional Autologous Full-Thickness Skin Grafting. Adv. Wound Care.

[14] Singer A.J., Tassiopoulos A., Kirsner R.S. (2017). Evaluation and Management of Lower-Extremity Ulcers. N. Engl. J. Med..

[15] Rowan M.P., Cancio L.C., Elster E.A., Burmeister D.M., Rose L.F., Natesan S., Chan R.K., Christy R.J., Chung K.K. (2015). Burn wound healing and treatment: Review and advancements. Crit. Care.

[16] Reinke J.M., Sorg H. (2012). Wound repair and regeneration. Eur. Surg. Res..

[17] Ramasubbu D.A., Smith V., Hayden F., Cronin P. (2017). Systemic antibiotics for treating malignant wounds. Cochrane Database Syst. Rev..

[18] Brumberg V., Astrelina T., Malivanova T., Samoilov A. (2021). Modern wound dressings: Hydrogel dressings. Biomedicines.

[19] Barillo D.J., Barillo A.R., Korn S., Lam K., Attar P.S. (2017). The antimicrobial spectrum of Xeroform^®^. Burns.

[20] Fadilah N.I.M., Riha S.M., Mazlan Z., Wen A.P.Y., Hao L.Q., Joseph B., Maarof M., Thomas S., Motta A., Fauzi M.B. (2023). Functionalised-biomatrix for wound healing and cutaneous regeneration: Future impactful medical products in clinical translation and precision medicine. Front. Bioeng. Biotechnol..

[21] Kumar A., Jaiswal M. (2016). Design and in vitro investigation of nanocomposite hydrogel based in situ spray dressing for chronic wounds and synthesis of silver nanoparticles using green chemistry. J. Appl. Polym. Sci..

[22] Tavakoli S., Klar A.S. (2020). Advanced hydrogels as wound dressings. Biomolecules.

[23] Bilici C., Can V., Nöchel U., Behl M., Lendlein A., Okay O. (2016). Melt-processable shape-memory hydrogels with self-healing ability of high mechanical strength. Macromolecules.

[24] Md Fadilah N.I., Khairul Nizam N.A.A., Fauzi M.B. (2024). Antibacterial compounds-incorporated functional biomaterials for chronic wound healing application via 3D bioprinting: The mechanism of action. Int. J. Bioprinting.

[25] Akbar A.R., Su S., Amjad B., Cai Y., Lin L. (2018). Effect of Bamboo Viscose on the Wicking and Moisture Management Properties of Gauze. IOP Conf. Ser. Mater. Sci. Eng..

[26] Fadilah N.I.M., Ahmat N., Hao L.Q., Maarof M., Rajab N.F., Idrus R.B.H., Fauzi M.B. (2023). Biological Safety Assessments of High-Purified Ovine Collagen Type I Biomatrix for Future Therapeutic Product: International Organisation for Standardisation (ISO) and Good Laboratory Practice (GLP) Settings. Polymers.

[27] Sun M., Sun X., Wang Z., Guo S., Yu G., Yang H. (2018). Synthesis and properties of gelatin methacryloyl (GelMA) hydrogels and their recent applications in load-bearing tissue. Polymers.

[28] Firlar I., Altunbek M., McCarthy C., Ramalingam M., Camci-Unal G. (2022). Functional Hydrogels for Treatment of Chronic Wounds. Gels.

[29] Ko J.H., Yin H., An J., Chung D.J., Kim J.H., Lee S.B., Pyun D.G. (2010). Characterization of cross-linked gelatin nanofibers through electrospinning. Macromol. Res..

[30] Vhora I., Patil S., Bhatt P., Misra A. (2015). Protein– and Peptide–Drug Conjugates: An Emerging Drug Delivery Technology.

[31] Dias J.R., Baptista-Silva S., de Oliveira C.M.T., Sousa A., Oliveira A.L., Bártolo P.J., Granja P.L. (2017). In situ crosslinked electrospun gelatin nanofibers for skin regeneration. Eur. Polym. J..

[32] Ndlovu S.P., Ngece K., Alven S., Aderibigbe B.A. (2021). Gelatin-based hybrid scaffolds: Promising wound dressings. Polymers.

[33] Jia X., Kiick K.L. (2009). Hybrid multicomponent hydrogels for tissue engineering. Macromol. Biosci..

[34] Sohail M., Mudassir, Minhas M.U., Khan S., Hussain Z., de Matas M., Shah S.A., Khan S., Kousar M., Ullah K. (2019). Natural and synthetic polymer-based smart biomaterials for management of ulcerative colitis: A review of recent developments and future prospects. Drug Deliv. Transl. Res..

[35] Fadilah N.I.M., Maarof M., Motta A., Tabata Y., Fauzi M.B. (2022). The Discovery and Development of Natural-Based Biomaterials with Demonstrated Wound Healing Properties: A Reliable Approach in Clinical Trials. Biomedicines.

[36] Peers S., Montembault A., Ladavière C. (2020). Chitosan hydrogels for sustained drug delivery. J. Control. Release.

[37] Tudoroiu E.E., Dinu-Pîrvu C.E., Kaya M.G.A., Popa L., Anuța V., Prisada R.M., Ghica M.V. (2021). An overview of cellulose derivatives-based dressings for wound-healing management. Pharmaceuticals.

[38] Abazari M.F., Gholizadeh S., Karizi S.Z., Birgani N.H., Abazari D., Paknia S., Derakhshankhah H., Allahyari Z., Amini S.M., Hamidi M. (2021). Recent advances in cellulose-based structures as the wound-healing biomaterials: A clinically oriented review. Appl. Sci..

[39] Aderibigbe B.A., Buyana B. (2018). Alginate in wound dressings. Pharmaceutics.

[40] Varaprasad K., Jayaramudu T., Kanikireddy V., Toro C., Sadiku E.R. (2020). Alginate-based composite materials for wound dressing application:A mini review. Carbohydr. Polym..

[41] Barnett S.E., Varley S.J. (1987). The effects of calcium alginate on wound healing. Ann. R. Coll. Surg. Engl..

[42] Yang J.S., Xie Y.J., He W. (2011). Research progress on chemical modification of alginate: A review. Carbohydr. Polym..

[43] Price R.D., Myers S., Leigh I.M., Navsaria H.A. (2005). The Role of Hyaluronic Acid in Wound Healing. Am. J. Clin. Dermatol..

[44] Castrejón-Comas V., Alemán C., Pérez-Madrigal M.M. (2023). Multifunctional conductive hyaluronic acid hydrogels for wound care and skin regeneration. Biomater. Sci..

[45] Luo Z., Wang Y., Xu Y., Wang J., Yu Y. (2023). Modification and crosslinking strategies for hyaluronic acid-based hydrogel biomaterials. Smart Med..

[46] Pepe A., Laezza A., Armiento F., Bochicchio B. (2024). Chemical Modifications in Hyaluronic Acid-Based Electrospun Scaffolds. Chempluschem.

[47] Salleh A., Mustafa N., Teow Y.H., Fatimah M.N., Khairudin F.A., Ahmad I., Fauzi M.B. (2022). Dual-Layered Approach of Ovine Collagen-Gelatin/Cellulose Hybrid Biomatrix Containing Graphene Oxide-Silver Nanoparticles for Cutaneous Wound Healing: Fabrication, Physicochemical, Cytotoxicity and Antibacterial Characterisation. Biomedicines.

[48] Jridi M., Bardaa S., Moalla D., Rebaii T., Souissi N., Sahnoun Z., Nasri M. (2015). Microstructure, rheological and wound healing properties of collagen-based gel from cuttlefish skin. Int. J. Biol. Macromol..

[49] Haghi A.K., Oluwafemi O.S., Jose J.P., Maria H.J. (2013). Composites and Nanocomposites.

[50] Gaharwar A.K., Peppas N.A., Khademhosseini A. (2014). Nanocomposite hydrogels for biomedical applications. Biotechnol. Bioeng..

[51] Zhao F., Yao D., Guo R., Deng L., Dong A., Zhang J. (2015). Composites of polymer hydrogels and nanoparticulate systems for biomedical and pharmaceutical applications. Nanomaterials.

[52] Bahati D., Bricha M., El Mabrouk K. (2023). Synthesis, characterization, and in vitro apatite formation of strontium-doped sol-gel-derived bioactive glass nanoparticles for bone regeneration applications. Ceram. Int..

[53] Chelu M., Musuc A.M. (2023). Advanced Biomedical Applications of Multifunctional Natural and Synthetic Biomaterials. Processes.

[54] Md Fadilah N.I., Rahman M.B.A., Yusof L.M., Mustapha N.M., Ahmad H. (2021). The therapeutic effect and in vivo assessment of palmitoyl-gdph on the wound healing process. Pharmaceutics.

[55] Song Y., Wu C., Zhang X., Bian W., Liu N., Yin S., Yang M.F., Luo M., Tang J., Yang X. (2019). A short peptide potentially promotes the healing of skin wound. Biosci. Rep..

[56] Amantana A., Moulton H.M., Cate M.L., Reddy M.T., Whitehead T., Hassinger J.N., Youngblood D.S., Iversen P.L. (2007). Pharmacokinetics, biodistribution, stability and toxicity of a cell-penetrating peptide—Morpholino oligomer conjugate. Bioconjug. Chem..

[57] Yang Z., Peng H., Wang W., Liu T. (2010). Crystallization behavior of poly(ε-caprolactone)/layered double hydroxide nanocomposites. J. Appl. Polym. Sci..

[58] Mondal J.H., Ahmed S., Das D. (2014). Physicochemical analysis of mixed micelles of a viologen surfactant: Extended to water-in-oil (w/o) microemulsion and cucurbit[8]uril-assisted vesicle formation. Langmuir.

[59] Das S., Das D. (2021). Rational Design of Peptide-based Smart Hydrogels for Therapeutic Applications. Front. Chem..

[60] Aggeli A. (1997). Engineering of peptide β-sheet nanotapes. J. Mater. Chem..

[61] Estroff L.A., Hamilton A.D. (2004). Water gelation by small organic molecules. Chem. Rev..

[62] Guan T., Li J., Chen C., Liu Y. (2022). Self-Assembling Peptide-Based Hydrogels for Wound Tissue Repair. Adv. Sci..

[63] Fadilah N.I.M., Ahmad H., Abdul Rahman M.B., Chia S.L., Ng S.F., Leong S.W. (2020). Synthesis and in vitro biological evaluations of novel tetrapeptide as therapeutic agent for wound treatment. J. Saudi Chem. Soc..

[64] Edwards-Gayle C.J.C., Hamley I.W. (2017). Self-assembly of bioactive peptides, peptide conjugates, and peptide mimetic materials. Org. Biomol. Chem..

[65] Zou P., Chen W.T., Sun T., Gao Y., Li L.L., Wang H. (2020). Recent advances: Peptides and self-assembled peptide-nanosystems for antimicrobial therapy and diagnosis. Biomater. Sci..

[66] Nur Izzah M.F., Ahmad H., Rahman M.F.A., Rahman N.A. (2019). Electrospun poly (vinyl alcohol) nanofibers doped with mesoporous silica nanoparticles for controlled release of hydrophilic model drug. Malaysian J. Anal. Sci..

[67] Silva G.A., Czeisler C., Niece K.L., Beniash E., Harrington D.A., Kessler J.A., Stupp S.I. (2004). Selective Differentiation of Neural Progenitor Cells by High-Epitope Density Nanofibers. Science.

[68] Chen Y., Wang X., Tao S., Wang Q., Ma P.Q., Li Z.B., Wu Y.L., Li D.W. (2023). Research advances in smart responsive-hydrogel dressings with potential clinical diabetic wound healing properties. Mil. Med. Res..

[69] Stefanov I., Pérez-Rafael S., Hoyo J., Cailloux J., Santana Pérez O.O., Hinojosa-Caballero D., Tzanov T. (2017). Multifunctional Enzymatically Generated Hydrogels for Chronic Wound Application. Biomacromolecules.

[70] Gavel P.K., Parmar H.S., Tripathi V., Kumar N., Biswas A., Das A.K. (2019). Investigations of Anti-Inflammatory Activity of a Peptide-Based Hydrogel Using Rat Air Pouch Model. ACS Appl. Mater. Interfaces.

[71] Gavel P.K., Dev D., Parmar H.S., Bhasin S., Das A.K. (2018). Investigations of Peptide-Based Biocompatible Injectable Shape-Memory Hydrogels: Differential Biological Effects on Bacterial and Human Blood Cells. ACS Appl. Mater. Interfaces.

[72] Acar H., Srivastava S., Chung E.J., Schnorenberg M.R., Barrett J.C., LaBelle J.L., Tirrell M. (2017). Self-assembling peptide-based building blocks in medical applications. Adv. Drug Deliv. Rev..

[73] Habibi N., Kamaly N., Memic A., Shafiee H. (2016). Self-assembled peptide-based nanostructures: Smart nanomaterials toward targeted drug delivery. Nano Today.

[74] Stephanopoulos N., Ortony J.H., Stupp S.I. (2013). Self-assembly for the synthesis of functional biomaterials. Acta Mater..

[75] Gavel P.K., Kumar N., Parmar H.S., Das A.K. (2020). Evaluation of a Peptide-Based Coassembled Nanofibrous and Thixotropic Hydrogel for Dermal Wound Healing. ACS Appl. Bio Mater..

[76] Eskandari S., Guerin T., Toth I., Stephenson R.J. (2017). Recent advances in self-assembled peptides: Implications for targeted drug delivery and vaccine engineering. Adv. Drug Deliv. Rev..

[77] Ferreira N.N., Ferreira L.M.B., Cardoso V.M.O., Boni F.I., Souza A.L.R., Gremião M.P.D. (2018). Recent advances in smart hydrogels for biomedical applications: From self-assembly to functional approaches. Eur. Polym. J..

[78] Barros S.C., Martins J.A., Marcos J.C., Cavaco-Paulo A. (2012). Influence of secretory leukocyte protease inhibitor-based peptides on elastase activity and their incorporation in hyaluronic acid hydrogels for chronic wound therapy. Biopolymers.

[79] Sedighi M., Shrestha N., Mahmoudi Z., Khademi Z., Ghasempour A., Dehghan H., Talebi S.F., Toolabi M., Chen B. (2023). Multifunctional Self-Assembled Peptide Hydrogels for Biomedical Applications. Polymers.

[80] Sirousazar M., Forough M., Farhadi K., Shaabani Y., Molaei R. (2014). Hydrogels: Properties, Preparation, Characterization and Biomedical, Applications in Tissue Engineering, Drug, Delivery and Wound Care. Advanced Healthcare Materials.

[81] Xie Z., Aphale N.V., Kadapure T.D., Wadajkar A.S., Orr S., Gyawali D., Qian G., Nguyen K.T., Yang J. (2015). Design of antimicrobial peptides conjugated biodegradable citric acid derived hydrogels for wound healing. J. Biomed. Mater. Res.-Part A.

[82] Thapa R.K., Diep D.B., Tønnesen H.H. (2020). Topical antimicrobial peptide formulations for wound healing: Current developments and future prospects. Acta Biomater..

[83] Kim S., Kim J.H., Lee J.S., Park C.B. (2015). Beta-Sheet-Forming, Self-Assembled Peptide Nanomaterials towards Optical, Energy, and Healthcare Applications. Small.

[84] Trier A.M., Mack M.R., Kim B.S. (2019). The Neuroimmune Axis in Skin Sensation, Inflammation, and Immunity. J. Immunol..

[85] Md Fadilah N.I., Mohd Abdul Kader Jailani M.S., Badrul Hisham M.A.I., Sunthar Raj N., Shamsuddin S.A., Ng M.H., Fauzi M.B., Maarof M. (2022). Cell secretomes for wound healing and tissue regeneration: Next generation acellular based tissue engineered products. J. Tissue Eng..

[86] Grek C.L., Prasad G.M., Viswanathan V., Armstrong D.G., Gourdie R.G., Ghatnekar G.S. (2015). Topical administration of a connexin43-based peptide augments healing of chronic neuropathic diabetic foot ulcers: A multicenter, randomized trial. Wound Repair Regen..

[87] Gelain F., Luo Z., Zhang S. (2020). Self-Assembling Peptide EAK16 and RADA16 Nanofiber Scaffold Hydrogel. Chem. Rev..

[88] Thota C.K., Berger A.A., Elomaa L., Nie C.X., Böttcher C., Koksch B. (2020). Coassembly Generates Peptide Hydrogel with Wound Dressing Material Properties. ACS OMEGA.

[89] Huang L.C., Wang H.C., Chen L.H., Ho C.Y., Hsieh P.H., Huang M.Y., Wu H.C., Wang T.W. (2019). Bioinspired Self-assembling Peptide Hydrogel with Proteoglycan-assisted Growth Factor Delivery for Therapeutic Angiogenesis. Theranostics.

[90] Yuan J.Z., Wang Y., Yang W.G., Li X., Tao K.S., He W.X., Yan J. (2023). Biomimetic peptide dynamic hydrogel inspired by humanized defensin nanonets as the wound-healing gel coating. Chem. Eng. J..

[91] Hao M., Ding C., Sun S., Peng X., Liu W. (2022). Chitosan/Sodium Alginate/Velvet Antler Blood Peptides Hydrogel Promotes Diabetic Wound Healing via Regulating Angiogenesis, Inflammatory Response and Skin Flora. J. Inflamm. Res..

[92] Chawla V., Sharma S., Singh Y. (2023). Yttrium Oxide Nanoparticle-Loaded, Self-Assembled Peptide Gel with Antibacterial, Anti-Inflammatory, and Proangiogenic Properties for Wound Healing. ACS Biomater. Sci. Eng..

[93] Qin P., Tang J., Sun D.D., Yang Y., Liu N.X., Li Y.L., Fu Z., Wang Y.L., Li C., Li X.J. (2022). Zn^2+^ Cross-Linked Alginate Carrying Hollow Silica Nanoparticles Loaded with RL-QN15 Peptides Provides Promising Treatment for Chronic Skin Wounds. ACS Appl. Mater. Interfaces.

[94] Lou P., Liu S., Wang Y., Pan C., Xu X., Zhao M., Liao G., Yang G., Yuan Y., Li L. (2021). Injectable self-assembling peptide nanofiber hydrogel as a bioactive 3D platform to promote chronic wound tissue regeneration. Acta Biomater..

[95] Shaik M.I., Sarbon N.M. (2022). A Review on Purification and Characterization of Anti-proliferative Peptides Derived from Fish Protein Hydrolysate. Food Rev. Int..

[96] Li J., Ma X.G., Wu D.G., Su Z.W., Su H., Liu Z.X., Chen Y., Yu B. (2024). Hybrid Self-Assembled Peptide Hydrogels Promote Skin Wound Healing in Diabetic Mice. ACS Appl. Polym. Mater..

[97] Wang L., Li J., Xiong Y., Wu Y.H., Yang F., Guo Y., Chen Z.L., Gao L.Q., Deng W.B. (2021). Ultrashort Peptides and Hyaluronic Acid-Based Injectable Composite Hydrogels for Sustained Drug Release and Chronic Diabetic Wound Healing. ACS Appl. Mater. Interfaces.

[98] Stojkov G., Niyazov Z., Picchioni F., Bose R.K. (2021). Relationship between structure and rheology of hydrogels for various applications. Gels.

[99] Wang W., Han R., Tang K., Zhao S., Ding C., Luo X. (2021). Biocompatible peptide hydrogels with excellent antibacterial and catalytic properties for electrochemical sensing application. Anal. Chim. Acta.

[100] Zulkiflee I., Fauzi M.B. (2021). Gelatin-polyvinyl alcohol film for tissue engineering: A concise review. Biomedicines.

[101] Hoque J., Prakash R.G., Paramanandham K., Shome B.R., Haldar J. (2017). Biocompatible injectable hydrogel with potent wound healing and antibacterial properties. Mol. Pharm..

[102] Kim J.E., Lee J.H., Kim S.H., Jung Y. (2017). Skin Regeneration with Self-Assembled Peptide Hydrogels Conjugated with Substance P in a Diabetic Rat Model. Tissue Eng. Part A.

[103] Stern D., Cui H.G. (2019). Crafting Polymeric and Peptidic Hydrogels for Improved Wound Healing. Adv. Healthc. Mater..

[104] Rijal N.P., Narmoneva D.A. (2020). Biomaterials for Diabetic Wound-Healing Therapies.

[105] Carrejo N.C., Moore A.N., Silva T.L.L., Leach D.G., Li I.C., Walker D.R., Hartgerink J.D. (2018). Multidomain Peptide Hydrogel Accelerates Healing of Full-Thickness Wounds in Diabetic Mice. ACS Biomater. Sci. Eng..

[106] Kang H.J., Chen N., Dash B.C., Hsia H.C., Berthiaume F. (2021). Self-Assembled Nanomaterials for Chronic Skin Wound Healing. Adv. Wound Care.

[107] López-Gutierrez J., Ramos-Payán R., Ayala-Ham A., Romero-Quintana J.G., Castillo-Ureta H., Villegas-Mercado C., Bermúdez M., Sanchez-Schmitz G., Aguilar-Medina M. (2023). Biofunctionalization of hydrogel-based scaffolds for vascular tissue regeneration. Front. Mater..

[108] Xiao Y., Reis L.A., Feric N., Knee E.J., Gu J.H., Cao S.W., Laschinger C., Londono C., Antolovich J., McGuigan A.P. (2016). Diabetic wound regeneration using peptide-modified hydrogels to target re-epithelialization. Proc. Natl. Acad. Sci. USA.

[109] Su Y., Wang H., Mishra B., Lakshmaiah Narayana J., Jiang J., Reilly D.A., Hollins R.R., Carlson M.A., Wang G., Xie J. (2019). Nanofiber Dressings Topically Delivering Molecularly Engineered Human Cathelicidin Peptides for the Treatment of Biofilms in Chronic Wounds. Mol. Pharm..

[110] Kim K., Mahajan A., Patel K., Syed S., Acevedo-Jake A.M., Kumar V.A. (2021). Materials and Cytokines in the Healing of Diabetic Foot Ulcers. Adv. Ther..

[111] Gao Y.F., Li Z., Huang J., Zhao M., Wu J. (2020). In situ formation of injectable hydrogels for chronic wound healing. J. Mater. Chem. B.

[112] Mandla S., Huyer L.D., Wang Y.F., Radisic M. (2019). Macrophage Polarization with Angiopoietin-1 Peptide QHREDGS. ACS Biomater. Sci. Eng..

[113] Wei Q., Duan J., Ma G., Zhang W., Wang Q., Hu Z. (2019). Enzymatic crosslinking to fabricate antioxidant peptide-based supramolecular hydrogel for improving cutaneous wound healing. J. Mater. Chem. B.

[114] Balaji S., Vaikunth S.S., Lang S.A., Sheikh A.Q., Lim F.Y., Crombleholme T.M., Narmoneva D.A. (2012). Tissue-engineered provisional matrix as a novel approach to enhance diabetic wound healing. Wound Repair Regen..

[115] Nidadavolu L.S., Stern D., Lin R., Wang Y.Z., Li Y., Wu Y.Q., Marin S., Antonio M.J., Yenokyan G., Boronina T. (2021). Valsartan nano-filaments alter mitochondrial energetics and promote faster healing in diabetic rat wounds. Wound Repair Regen..

[116] Wan T., Li L.C., Zhu Z.Y., Liu S.Y., Zhao Y.Q., Yu M.S. (2017). Scorpion Venom Active Polypeptide May Be a New External Drug of Diabetic Ulcer. Evid.-Based Complement. Altern. Med..

